# Molecular profiles of Quadriceps muscle in *myostatin*-null mice reveal PI3K and apoptotic pathways as myostatin targets

**DOI:** 10.1186/1471-2164-10-196

**Published:** 2009-04-27

**Authors:** Ilham Chelh, Bruno Meunier, Brigitte Picard, Mark James Reecy, Catherine Chevalier, Jean-François Hocquette, Isabelle Cassar-Malek

**Affiliations:** 1 INRA, UR1213, Unité de Recherches sur les Herbivores, Equipe Croissance et Métabolisme du Muscle, Theix, F-63122 Saint-Genès-Champanelle, France; 2 Iowa State University, Animal Science Dept, 2255 Kildee Hall, Ames, IA 50011-3150, USA; 3 Plateforme Puces à ADN, Ouest Génopole, U915, Institut du Thorax, Faculté de Médecine 1, rue Gaston Veil, 44035 Nantes cédex, France

## Abstract

**Background:**

Myostatin (MSTN), a member of the TGF-β superfamily, has been identified as a negative regulator of skeletal muscle mass. Inactivating mutations in the MSTN gene are responsible for the development of a hypermuscular phenotype. In this study, we performed transcriptomic and proteomic analyses to detect altered expression/abundance of genes and proteins. These differentially expressed genes and proteins may represent new molecular targets of MSTN and could be involved in the regulation of skeletal muscle mass.

**Results:**

Transcriptomic analysis of the Quadriceps muscles of 5-week-old *MSTN*-null mice (n = 4) and their controls (n = 4) was carried out using microarray (human and murine oligonucleotide sequences) of 6,473 genes expressed in muscle. Proteomic profiles were analysed using two-dimensional gel electrophoresis coupled with mass spectrometry. Comparison of the transcriptomic profiles revealed 192 up- and 245 down- regulated genes. Genes involved in the PI3K pathway, insulin/IGF pathway, carbohydrate metabolism and apoptosis regulation were up-regulated. Genes belonging to canonical Wnt, calcium signalling pathways and cytokine-receptor cytokine interaction were down-regulated. Comparison of the protein profiles revealed 20 up- and 18 down-regulated proteins spots. Knockout of the MSTN gene was associated with up-regulation of proteins involved in glycolytic shift of the muscles and down-regulation of proteins involved in oxidative energy metabolism. In addition, an increased abundance of survival/anti-apoptotic factors were observed.

**Conclusion:**

All together, these results showed a differential expression of genes and proteins related to the muscle energy metabolism and cell survival/anti-apoptotic pathway (e.g. DJ-1, PINK1, 14-3-3ε protein, TCTP/GSK-3β). They revealed the PI3K and apoptotic pathways as MSTN targets and are in favour of a role of MSTN as a modulator of cell survival in vivo.

## Background

Myostatin (MSTN), a member of the TGF-β superfamily, has emerged as a key factor of muscle development and maintenance of muscle mass homeostasis [1]. This has strengthened the idea that MSTN is a potential target for novel therapeutic strategies to improve the disease symptoms with potential long-term benefits in a variety of muscular dystrophies [2], or for improving the management of muscular growth in livestock animals [3].

Inactivating mutations of the MSTN gene are responsible for the development of a hypermuscular phenotype in cattle [4,5], dogs [6], mice [7] and humans [8]. The hypermuscular phenotype associated with constitutive MSTN loss-of-function results from both hyperplasia (increased number of fibers) and hypertrophy (increased size of individual fibers). An hypermuscular phenotype of the same magnitude as that observed for constitutive knockout has also been observed in mice with a conditional MSTN knockout (postnatal inactivation of MSTN) generated by a Cre-Lox system [9]. This demonstrated that MSTN modulates the muscle mass throughout development. The ability of MSTN to control myoblast cell proliferation and differentiation has been demonstrated. Examination of the molecular action of MSTN has revealed an inhibitory influence on proliferation through the control of cell cycle progression [10,11]. MSTN also inhibits myoblast differentiation [12] partially through a decreased expression of Myogenic Regulatory Factors (reviewed by [13]). Myogenin and p21CKI have been identified as the major physiological targets of endogenous MSTN in murine cells [14]. MSTN has also been shown to negatively regulate satellite cell activation and self-renewal [15] and this action may involve a regulation of Pax7 [16]. Furthermore, we recently demonstrated that MSTN can regulate satellite cell proliferation via regulation of WNT4 [17].

Recent data established that MSTN induces muscle atrophy. In mice, muscle disuse-induced atrophy generated by hindlimb unloading is associated with a reversible increase in MSTN mRNA abundance [18]. MSTN has been implicated in muscle wasting in various diseases (HIV, cachexia, cancer, Duchenne's muscular dystrophy) and ageing [19]. Transgenic mice that overexpress MSTN selectively in skeletal muscle have lower muscle mass [20]. In adult rats, ectopic MSTN expression induces atrophy of skeletal muscle as shown by a significant decrease in muscle mass, fiber cross-sectional area and protein content [21]. This is associated with decreased expression of genes structural proteins (myosin heavy chain IIb, troponin I and desmin) and myogenic transcription factors. Moreover, inhibition of MSTN rescues the muscular atrophy of caveolin-3-deficient mice [22] and protects against muscle atrophy due to glucocorticoid treatment [23]. If some of the mechanisms by which MSTN contributes to atrophy have been clarified, e.g. FOXO1 activation and subsequent activation of ubiquitin proteolytic system [24], the relative contribution of MSTN to the regulation of the balance between atrophic and hypertrophic processes in muscle remains to be further elucidated. Some questions remain to be answered such as the possibility that MSTN might inhibit muscle hypertrophy rather than induce atrophy [24,25].

We have previously analysed the metabolic profile of double-muscled cattle [26] and more recently the molecular profiles in muscle in the context of MSTN loss-of-function [27,28] and hypothesised that these should help in the understanding of molecular mechanisms by which MSTN regulates muscle mass homeostasis. The main objective of the present study was to identify MSTN targets involved in the regulation of muscle mass. For this, we examined the transcriptional and proteomic profiles in the *Quadriceps *muscle of *MSTN*-null mice and their control littermates. The originality of this work was to combine two genomic approaches, namely transcriptomics and proteomics, providing complementary data.

## Results

### Transcriptomic profiles in *myostatin*-null mice muscle

To search for novel candidate MSTN targets, we used oligonucleotide microarrays (human and murine oligonucleotide sequences) of 6,473 genes expressed in muscle. We examined mRNA expression in individual samples from 4 constitutive *MSTN*-null and 4 control littermates. A reference sample allowing hybridization with detection of 57 to 68% reliable spots was used to compare the patterns of muscle gene expression between groups of mice. The hybridization results showed that, among 6,473 genes represented on the chip, an average of 2,496 (39%) gave valid expression values. Significance analysis of microarray (SAM) was conducted to detect statistically significant expression changes. In total, SAM analysis revealed the differential expression of 192 up- and 245 down- regulated genes (FDR <5%, see additional files 1 and 2).

Bioinformatic analysis indicated that genes belonging to the Phosphatidyl Inositol 3-kinase (PI3K) pathway, insulin/IGF pathway protein kinase β signalling, carbohydrate metabolism, cell differentiation were up-regulated (Table 1). Conversely, genes belonging to the canonical Wnt signalling pathway, calcium signalling pathway and cytokine-cytokine receptor interaction were down-regulated (Table 1). Twenty-three of the differentially expressed genes encoded for transcription factors of which 8 were up-regulated (e.g. foxa3) and 15 were down-regulated (e.g. Lef1).

**Table 1:** Distribution of differential genes in *myostatin*-null mice *vs *control mice according to KEGG pathways.

**#Entity KEGG**	**Down regulated genes**	**N =**	**Up regulated genes**	**N =**	**Unadj. P value**	**Adj. P value**
**Phosphatidylinositol signaling system**	No genes	0	Inppl1, PIK3R3, PIK3CG PLCG1			
**Insulin signaling pathway**	Gsk3b	1	Ppp1cb, PTPRF, PIK3R3, PIK3CG, PRKAR1B	5	1.05E-01	1
**mTOR signaling pathway**	No genes	0	MO25, PIK3R3, PIK3CG	3	1.09E-01	1
**Chronic myeloid leukemia**	No genes	0	PIK3R3, PIK3CG, Abl1	3	1.09E-01	1
**T cell receptor signaling pathway**	No genes	0	PIK3R3, PIK3CG, PLCG1	3	1.09E-01	1
**Fructose and mannose metabolism**	Akr1e1, PMM2, Khk, OGT	4	No genes	0	1.19E-01	1
**ECM-receptor interaction**	LAMC3, Col4a2, COL6A1, ITGB8, ITGA9	5	Col4a4	1	2.08E-01	1
**Calcium signaling pathway**	PDE1B, Camk2a, CAMK4, Cacna1i, HTR2A, ADORA2A	6	PLCG1, Ptgfr	2	2.74E-01	1
**Regulation of actin cytoskeleton**	Limk1, ARPC4 ITGB8, ITGA9	4	FGF6, Ppp1cb, PIK3R3, PIK3CG, CFL2, Actb, MYL1	7	3.50E-01	1
**MAPK signaling pathway**	Cacna1i	1	MAP2K7, FGF6, PLA2G6	3	3.51E-01	1
**Natural killer cell mediated cytotoxicity**	KLRC2	1	PIK3R3, PIK3CG, PLCG1	3	3.51E-01	1
**Apoptosis**	Atm	1	PIK3R3, PIK3CG, PRKAR1B	3	3.51E-01	1
**Wnt signaling pathway**	Gsk3b, LRP5, Camk2a, LEF1	4	CCND3	1	3.65E-01	1
**Leukocyte transendothelial migration**	Cxcl15, Cxcl13	2	PIK3R3, PIK3CG, Actb, PLCG1	4	4.27E-01	1
**Cytokine-cytokine receptor interaction**	IL23A Cxcl15, CXCR3, IL17, Cxcl13	5	CCL22, CCR7	2	4.40E-01	1
**Toll-like receptor signaling pathway**	Irf3	1	PIK3R3, PIK3CG	2	6.09E-01	1
**B cell receptor signaling pathway**	Gsk3b	1	PIK3R3, PIK3CG	2	6.09E-01	1
**GnRH signaling pathway**	Camk2a	1	MAP2K7, PLA2G6	2	6.09E-01	1
**Axon guidance**	Limk1, Gsk3b, Epha2	3	SEMA3A, Abl1, CFL2, EFNA4	4	7.10E-01	1
**Focal adhesion**	LAMC3, Gsk3b, Col4a2, COL6A1, ITGB8, ITGA9	6	CCND3, Ppp1cb, PIK3R3, PIK3CG, Actb, Col4a4	6	1	1
**Hedgehog signaling pathway**	Gsk3b	1	Gas1	1	1	1
**Alzheimer's disease**	Gsk3b, Apoe	2	LRP1	1	1	1

N: Number of genes involved in each biological process; Unadj. P value: Unadjusted P value, P value from Fisher's exact test without adjusting for multiple comparisons; Adj. P value: adjusted P value, after correction for multiple testing.

Expression of upstream components of the PI3K survival pathway (Akt, GSK-3β) were surveyed. The steady-state level of Akt mRNA (Table 1) and phosphorylated protein (Table 2) were increased in the muscles of *MSTN*-null mice. Differential expression of the GSK-3β was confirmed by qPCR and western-blot (Table 2) analyses. In addition, the ratio of serine-9 phosphorylated GSK-3β/GSK-3β ration was increased, which illustrates GSK-3β activation in *MSTN*-null versus wild-type skeletal muscle (Table 2).

**Table 2:** Differential expression of myostatin targets genes confirmed by qPCR and by western-blot experiments in MSTN-null mice vs their control littermates.

	**Proteomics**	**Transcriptomics**	**qPCR**	**Western-Blot**
**H-FABP**	-2.1			-9.1
**MYBPH**	3.1			1.4
**MyHCII**				4.9
**Syntrophin A**	1.5			2.8

**p-Akt**				2.2
**GSK-3β**		-1.9	-2.4	
**GSK-3β(ser9)/GSK-3β**				1.7 t
**PI3KR3**		1.1	1.8	

**DJ-1**	1.5		1.7	2.2
**PINK1**		1.9		3.4
**PTEN**			-1.6 t	-1.6

**Mortalin**	-1.5		-1.4	-2.1
**TCTP**	2.7			1.6
**14-3-3E**	1.8		1.4	1.7

**mfgf6**			1.9	

Results are expressed as Fold Changes and all the differences have been declared significant (P < 0.05) except for some tendencies (t: P < 0.10).

H-FABP: Heart-Fatty acid binding protein H; MyBP-H: Myosin binding protein H; MyHCII: fast myosin heavy chain isoform; pAkt: phosphorylated Akt; GSK-3β: Glycogen synthase kinase 3β; GSK-3β (ser9): Glycogen synthase kinase 3β (phosphorylated at serine 9); PI3KR3: Phosphatidylinositol 3-kinase regulatory subunit; DJ-1: cancer- and Parkinson's disease-associated protein (park7); PINK1: PTEN-induced putative kinase 1; PTEN: tumour-suppressor phosphatase with tensin homology. TCTP: Translationally controlled tumor protein; 14-3-3E: 14-3-3ε protein; mfgf6: murine Fibroblast growth factor 6.

### Proteomic profiles in *myostatin*-null mice muscle

We have used two-dimensional polyacrylamide gel electrophoresis, high-throughput image analysis and candidate picking, coupled with mass spectrophotometry to investigate the proteome profile of *MSTN*-null mice *vs *control littermates. Comparison of the protein profiles using SAM (FDR <5%) revealed the differential abundance of 38 protein spots (20 up-regulated and 18 down-regulated) in *MSTN*-null mice *vs *wild-type controls. Mass spectrometry analyses allowed the identification of the candidate spots (Table 3). Differential expression was confirmed for some selected proteins by western-blot analysis (Table 2).

**Table 3:** Differentially expressed muscle proteins in *myostatin*-null mice *vs *control mice.

**Spot no.**	**Protein name**	**Relevant Swiss Prot Accession number**	**pI**	**Mr (kDa)**	**FC**
1781	**Eef1g protein [Mus musculus]**	Q8R1N8_MOUSE	7.02	37.178	Increased 2.8
1735	**Adenylate kinase 1 [Mus musculus]**	KAD1_MOUSE	5.67	21.64	Increased 1.5
1728	**DJ-1 protein [Mus musculus]**	PARK7_MOUSE	6.32	20.236	Increased 1.5
1690	**NADH dehydrogenase (ubiquinone) Fe-S protein 3 [Mus musculus]**	NDUS3_MOUSE	6.67	30.302	Increased 1.7
1690	**Guanidinoacetate methyltransferase [Mus musculus]**	GAMT_MOUSE	5.43	26.604	Increased 1.7
1663	**Capping protein (actin filament) muscle Z-line, beta isoform a [Mus musculus]**	A2AMV4_MOUSE	5.47	31.611	Increased 1.4
1593	**Capping protein (actin filament) muscle Z-line, alpha 2 [Mus musculus]**	Q3UBZ3_MOUSE	5.57	33.118	Increased 1.4
1673	**Muscle glycogen phosphorylase [Mus musculus]**	PYGM_MOUSE	6.65	97.681	Increased 2.4
1894	**Glycogen phosphorylase, muscle form (EC 2.4.1.1) (Myophosphorylase) [Mus musculus]**	PYGM_MOUSE	6.65	97.094	Increased 1.7
1393	**Enolase 1, alpha non-neuron [Mus musculus]**	ENOA_MOUSE	6.37	47.453	Increased 1.3
1230	**t-complex protein 1 [Mus musculus]**	TCPA1_MOUSE	5.82	60.867	Increased 1.7
1164	**Myosin binding protein H [Mus musculus]**	MYBPH_MOUSE	6.21	53.065	Increased 3.1
1166	**Myosin binding protein H [Mus musculus]**	MYBPH_MOUSE	6.09	53.065	Increased 1.5
1731	**Translationally-controlled tumor protein (TCTP) (p23) (21 kDa polypeptide) (p21) [Mus musculus]**	TCTP_MOUSE	4.76	19.45	Increased 2.7
1703	**14-3-3 protein epsilon (14-3-3E) [Mus musculus]**	1433E_MOUSE	4.63	29.155	Increased 1.8
1706	**Calpain small subunit 1 (CSS1) (Calcium-dependent protease small subunit 1) [Mus musculus]**	CPNS1_MOUSE	5.41	28.445	Increased 1.4
1718	**Proteasome subunit beta type 4 precursor (EC 3.4.25.1) (Proteasome beta chain) [Mus musculus]**	PSB4_MOUSE	5.47	29.097	Increased 1.4
1980	**Purine nucleoside phosphorylase (EC 2.4.2.1) (Inosine phosphorylase) (PNP) [Mus musculus]**	PNPH_MOUSE	5.78	32.256	Increased 2.9
1427	**Alpha-1-syntrophin [Mus musculus]**	SNTA1_MOUSE	6.4	53.632	Increased 1.5
1804	**Fatty acid binding protein 3, muscle and heart [Mus musculus]**	FABPH_MOUSE	5.75	14.81	Decreased 2.1
1803	**Fatty acid binding protein 3, muscle and heart [Mus musculus]**	FABPH_MOUSE	6.11	14.81	Decreased 2.1
1764	**heat shock protein, alpha-crystallin-related, B6 [Mus musculus]**	HSPB6_MOUSE	5.64	17.567	Decreased 1.8
1620	**Pyruvate dehydrogenase (lipoamide) beta [Mus musculus]**	ODPB_MOUSE	6.41	39.254	Decreased 1.4
1571	**Lactate dehydrogenase 2, B chain [Mus musculus]**	LDHB_MOUSE	5.7	36.834	Decreased 2.4
1541	**Isocitrate dehydrogenase 3 (NAD+) alpha [Mus musculus]**	IDH3A_MOUSE	6.27	40.069	Decreased 1.3
1534	**Isocitrate dehydrogenase 3 (NAD+) alpha [Mus musculus]**	IDH3A_MOUSE	6.27	40.069	Decreased 1.6
1407	**Ubiquinol-cytochrome c reductase core protein 1 [Mus musculus]**	QCR1_MOUSE	5.81	53.446	Decreased 1.3
1373	**ATP synthase, H+ transporting mitochondrial F1 complex, beta subunit [Mus musculus]**	AT5F1_MOUSE	5.19	56.265	Decreased 2.3
1178	**Dihydrolipoamide S-acetyltransferase (E2 component of pyruvate dehydrogenase complex) [Mus musculus]**	ODP2_MOUSE	8.81	68.473	Decreased 1.5
1084	**Heat shock protein 9A [Mus musculus]**	GRP75_MOUSE	5.91	73.768	Decreased 1.5
1691	**NADH-ubiquinone oxidoreductase 30 kDa subunit, mitochondrial precursor [Mus musculus]**	NUGM_MOUSE	6.4	30.189	Decreased 1.3
1139	**Serum albumin precursor [Mus musculus]**	ALBU_MOUSE	5.75	68.648	Decreased 1.3
1109	**Serum albumin precursor [Mus musculus]**	ALBU_MOUSE	5.75	68.648	Decreased 1.6
1739	**ATP synthase D chain, mitochondrial [Mus musculus]**	ATP5H_MOUSE	5.52	18.607	Decreased 1.3
1152	**Succinate dehydrogenase [ubiquinone] flavoprotein subunit, mitochondrial precursor [Mus musculus]**	DHSA_MOUSE	7.06	72.539	Decreased 1.7
1400	**Ubiquinol-cytochrome-c reductase complex core protein I, mitochondrial precursor [Mus musculus]**	UQCR1_MOUSE	5.75	52.735	Decreased 1.5
1608	**Troponin T, fast skeletal muscle (TnTf) (Fast skeletal muscle troponin T) (fTnT) [Mus musculus]**	TNNT3_MOUSE	5.26	32.09	Decreased 1.6

Accession number: reference of each protein in Swiss-Prot database; pI: isoelectric point; Mr (kDa): molecular weight; FC: Fold-Change

Bioinformatic analysis indicated that most of the differential proteins were related to muscle energy metabolism. Enzymes of the citrate cycle (e.g. pyruvate dehydrogenase lipoamide, dihydrolipoamide S-acetyl transferase E2, isocitate dehydrogenase, Succinate dehydrogenase) and the respiratory chain (e.g. ATP synthase F1 beta, ATP synthase D chain, ubiquinol cytochrome c reductase core protein 1, NADH-ubiquinone oxidoreductase), and the heart-fatty acid binding protein (H-FABP) were down-regulated (Table 2; Table 3). Conversely two fragments of Glycogen phosphorylase, and enolase 1 (an enzyme of the glycolysis/gluconeogenesis pathway) were up-regulated (Table 3). Abundance levels of Guanidinoacetate methyltransferase, which catalyzes the last step of creatine biosynthesis, purine nucleoside phosphorylase, which catalyzes the reversible cleavage of inosine and guanosine to their respective bases hypoxanthine and guanine, and the adenylate kinase 1, which is an important enzyme in cellular adenine nucleotide homeostasis, were also increased. Myosin-binding protein H, which is a protein involved in the contractile apparatus, was revealed to be up-regulated in *MSTN*-null mice *vs *wild-type controls and this was confirmed by western-blot analysis (Table 2). Fast glycolytic Myosin Heavy chain isoforms (MyHCII) were also more abundant in *MSTN*-null mice vs wild-type controls in western-blot experiments (Table 2). α1-Syntrophin was found to be up-regulated in *MSTN*-null mice (Table 2; Table 3). All together, these data are representative of greater fast glycolytic metabolism in the muscle of *MSTN*-null mice.

**Figure 1:**
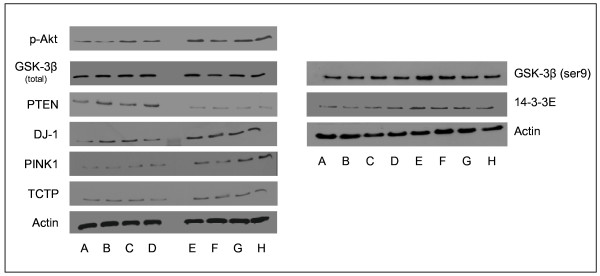
**Abundance of candidate proteins to be myostatin targets as revealed by western-blotting in the muscles of control and MSTN-null mice**. A-D: control mice; E-H: MSTN-null mice. pAkt: phosphorylated akt; GSK-3β: Glycogen synthase kinase 3β; GSK-3β (ser9): Glycogen synthase kinase 3β (phosphorylated at serine 9); DJ-1: cancer- and Parkinson's disease-associated protein (park7); PINK1: PTEN-induced putative kinase 1; PTEN: tumour-suppressor phosphatase with tensin homology. TCTP: Translationally controlled tumor protein; 14-3-3E: 14-3-3ε protein. Actin was used as a loading control protein.

A differential abundance of survival/mortality factors and some chaperones (Table 3) was also detected with the down regulation of Hsp alpha crystallin-related B6, Hsp 9A (mortalin), and the up-regulation of Tcp1, TCTP, DJ-1, and 14-3-3E. The differential abundance levels were confirmed by western-blot analyses for mortalin, DJ-1, TCTP and 14-3-3E (Table 2 and Figure 1). All together these data indicate that *myostatin *inactivation was associated with up-regulation of survival/anti-apoptotic factors, in favour to increased cell survival.

## Discussion

In this study, we have looked for novel myostatin targets and mechanisms involved in the control of muscle homeostasis. For this, we have used a functional genomics strategy, which combined both transcriptomic and proteomic profiling of *MSTN*-null *vs *wild-type control skeletal muscle. We hypothesized that examination of molecular profiles in the muscles of young growing mice in the context of MSTN loss-of-function would allow the identification of myostatin downstream targets. However any changes in gene and protein expression may also reflect consequences of loss of MSTN function. Anyway, this approach should give information on direct or indirect MSTN targets by revealing altered physiological pathways. A similar percentage of genes (17.5%) and proteins (15.3%) were declared to be differentially expressed with both techniques as indicated by the ratio of the number of differential genes and proteins to the total number of genes spotted on chips and proteins detected in 2-DE gels in the range of pH 4–7, respectively. Transcriptomics and proteomics provided complementary data and led us to analyse both gene expression by qPCR and protein abundance by western blotting for some candidate targets.

As expected, the expression of several genes and abundance of proteins involved in muscle energy metabolism were altered. Specifically we found an up-regulation of those involved in glycolytic metabolism and a down-regulation of mitochondrial proteins. Similar to previously published results [27-29], this provided additional evidence of a glycolytic shift within skeletal muscle in absence of functional myostatin. Additionally, expression level of α1-syntrophin, a predominant syntrophin isoform in skeletal and cardiac muscle, significantly increased in *MSTN*-null mice. This result is in agreement with the work of Vandebrouck and co-workers [30], which indicates that α1-syntrophin might play a potential signalling role in the regulation of calcium influx in muscle cells and thereby regulate muscle activity.

### Increased potential for PI3K pathway

Our study revealed that upstream components of the PI3K/Akt/GSK-3β, a signalling network that promotes cell survival and proliferation, were targets of myostatin inactivation. The core of the PI3K signalling pathway begins with PI3K activation and subsequent generation of the second messenger lipid phosphatidyl (3,4,5) triphosphate, which recruits and activates PI-dependent kinase. The latter phosphorylates and activates protein kinase B/Akt [31]. GSK-3β is a substrate for phosphorylated Akt and is inactivated upon phosphorylation. We detected the differential expression of mRNAs that encode for PIK3R3, PIK3CG, PLCG1, and GSK-3β. Increases in the abundance of phophorylated-Akt and in the activation ratio of GSK-3β (phosphorylated GSK-3β at serine 9/GSK-3β) were also detected (Table 2). This finding is original since the PI3K pathway is mainly regulated through post-translational modification namely phosphorylation. Thus, our results indicate that myostatin inactivation resulted in increased potential for PI3K/Akt activation and decreased potential for GSK-3β activity both at the transcriptional and post-translational levels.

Interestingly, we also detected the differential abundance of factors regulating the PI3K pathway e.g. DJ-1, PTEN and PINK1. DJ-1, a cancer- and Parkinson's disease-associated protein also called park7, protects cells from toxic stresses and is implicated in mitochondria and oxidative stress-related survival pathways (reviewed in [32]). It associates with chaperonnes including mortalin. DJ-1 has also been reported to be a novel regulator of the PI3K-PTEN pathway [33]. The tumour-suppressor phosphatase with tensin homology (PTEN) is the most important negative regulator of the cell-survival signalling initiated by PI3K (Figure 1). DJ-1 interacts with PTEN-induced putative kinase 1 (PINK1), which encodes a Ser/Thr kinase with a mitochondrial-targeting signal, and collaborate to protect cells against oxidative stress induced cell death [34]. Interestingly, our data show up-regulation of DJ-1 and PINK1 and down-regulation of PTEN both at the transcriptional and protein levels and this could account for activation of the PI3K pathway in *MSTN*-null mice.

The PI3K/Akt pathway plays a pivotal role in the promotion of cell proliferation and inhibition of cell death. It plays an important role in at least two signal transductory systems, namely, the Wnt/wingless and PI3K pathways, which influence proliferation and cell survival, respectively. Our data agree well with data that demonstrate a correlation between activation of PI3K pathway and increase in cell size [35] and the activation of Akt, which stimulates muscle hypertrophy and antagonizes the loss of muscle protein [36]. Akt signalling through GSK-3β, mTOR (a PI3K-related kinase) and Foxo1 was shown to be associated with skeletal muscle hypertrophy and atrophy processes in humans [37]. Recently, Amirouche and workers have shown that MSTN negatively regulates Akt/mTOR signalling pathway [38]. It was also shown that MSTN activates GSK-3β and decreases cyclin D1 by inhibiting the PI3K/Akt pathway and this has been proposed to be involved in the progression of muscle-disuse atrophy [39]. Lastly, the PI3K/Akt pathway is the major intracellular pathway activated by IGF-I stimulation during myogenesis [40]. A primary effect of GSK-3β inhibition in muscle is to promote myogenic differentiation and it has been proposed that IGF-mediated hypertrophy involves the negative regulation of GSK-3β activity [41].

### Altered expression/abundance of factors involved in cell survival/apoptosis

Our data revealed changes in the expression of genes and proteins related to cell survival/apoptosis pathways. Actually, we detected the down-regulation of some factors (e.g. alpha crystallin-related B6, heat shock protein 9A) and up-regulation of others (e.g. Dad1, survivin, TCTP, 14-3-3E) that may be a signature of increased cell survival and anti-apoptotic processes. Indeed, alpha crystallin-related B6, a heat shock protein 20, is thought to protect cells from apoptosis [42]. Heat shock protein 9A (Mortalin, Grp75), a member of the heat shock protein 70 family, plays a central role in mitochondrial import, energy generation and chaperoning of mis-folded proteins (for review [43]). This factor interacts with the tumor suppressor protein p53 and inactivates its transcriptional activation and apoptotic functions [44]. Up-regulation of mortalin contributes significantly to tumorigenesis [45]. Dad1 (defender against apoptotic death) has been reported to be a programmed cell death suppressor necessary for cell survival in a mammalian cell line [46] and the loss of Dad1 protein triggers apoptosis [47]. Survivin (Birc5) is a member of the inhibitor of apoptosis (IAP) family that prevents apoptotic cell death in various cell types including hematopoietic, endothelial, embryonic and neuronal cells [48]. Translationally controlled tumor protein (TCTP) is involved in the calcium binding and microtubule stabilization [49] and has been implicated in cell growth, cell cycle progression, malignant transformation and in protection of cells against various stress conditions and apoptosis [50]. 14-3-3E is a member of the 14-3-3 family of phosphoserine-binding adapter molecules that mediate a general survival-promoting function through interaction with target proteins, by enhancing pro-survival signalling while suppressing activity of pro-apoptotic proteins (reviewed by [51]). They are involved in the regulation of apoptosis through multiple interactions with proteins of the core mitochondrial machinery, pro-apoptotic transcription factors and their upstream signalling pathways. One prominent mechanism for the suppression of apoptosis is through 14-3-3-mediated sequestration of pro-apoptotic proteins (e.g. BAD, Bcl-2-antagonist of cell death; [52]). Furthermore, 14-3-3 proteins regulate the Akt/mTOR pathway via interaction with PRAS40, a mTOR binding partner [53]. In our study, 14-3-3E showed greater expression in the hypertrophied muscles of *MSTN*-null mice. Interestingly, some of the differential proteins detected in our study (e.g. DJ-1, T-complex protein 1, proteasome B chain) were reported to belong to the 14-3-3 interacting "phosphoproteome" [54].

Interestingly, in a previous study conducted in the context of MSTN loss of function in cattle [28], we have already found an up-regulation of genes encoding proteins involved in apoptosis (e.g DDX, PDCD6) and a down-regulation of anti-apoptotic genes belonging to the Bcl2 family. Supporting the hypothesis of decreased apoptosis in the muscles of *MSTN*-null mice, we found that the bioactive fragment of the initiator Caspase 8 was decreased in these mice compared to their littermates (data not shown). The functional significance of proteins related to cell survival/apoptosis to muscle hypertrophy remains to be further studied. However, the results of the present study suggest that MSTN may behave as an arbiter of the survival decision in the muscles. In *MSTN*-null mice, increased abundance of anti-apoptotic factors probably induces a by-pass of apoptosis and consequently increased cell survival, a process leading to hypertrophy (Figure 1). The involvement of MSTN in apoptosis remains scarce and controversial. There are conflicting results regarding MSTN and apoptosis. It is not clear whether this factor protects myoblasts from apoptosis [14,55] or promotes the apoptotic process [39,56]. This discrepancy may be explained by the different experimental conditions in which MSTN effect was examined *in vitro *(e.g. proliferation *vs *differentiation phase, myogenic cell lines *vs *primary myoblast cultures). Although skeletal muscle is considered to be rather resistant to apoptosis partly due to high concentration of endogenous inhibitors (reviewed in [57]), there is evidence that apoptotic events occur in skeletal muscle under physiological (exercise) and pathological conditions (e.g. muscle disuse-induced atrophy, denervation, ageing, dystrophinopathies) and may contribute to atrophy and loss of fibers [58-61]. "Myonuclear apoptosis" is a predominant form of apoptosis by which a loss of myonuclei occurs without cell death during muscle atrophy [62,63]. This process may have major consequence since (1) the number of myonuclei is a critical determinant for protein synthesis capacity (2) apoptosis within muscle may alter the myonuclear to cytosolic ratio (protein/DNA). Thus, modulation of myonuclear number or myonuclear domain size or both is a mechanism by which MSTN may contribute to muscle homeostasis. Inhibition of apoptosis in the context of MSTN loss-of-function could lead to increased myonucler survival and consequently to increased protein biosynthesis. Thus, this could contribute to the hypertrophic pathway, especially in growing mice. Work is progress to assay apoptotic process in muscles according to MSTN activity.

## Conclusion

**Figure 2:**
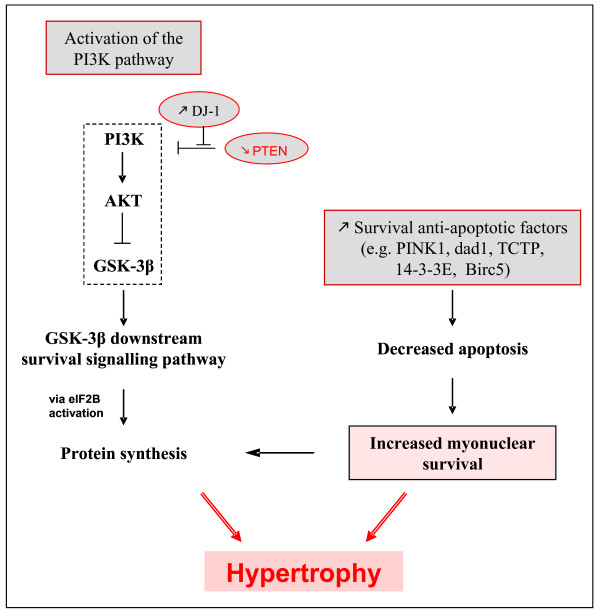
**In *MSTN*-null mice, regulators of the PI3K pathway and cell survival/apoptosis likely contribute to muscle hypertrophy**. *PI3K signalling pathway*: PI3K, Phosphatidyl Inositol 3 Kinase; Akt, protein kinase B; GSK-3β, Glycogen synthase kinase 3β; eIF2B, eukaryotic protein synthesis initiation factor 2B; PTEN, tumour-suppressor phosphatase with tensin homology (the most important negative regulator of the cell survival signalling initiated by PI3K); DJ-1, a cancer- and Parkinson's disease-associated protein (a novel regulator of the PI3K-PTEN pathway). *Survival/Anti-apoptotic factors*: PINK1, PTEN-induced putative kinase; Dad1, defender against apoptotic death; TCTP, Translationally controlled tumour protein; 14-3-3E, 14-3-3 protein epsilon; Birc5, survivin.

In conclusion, integration of transcriptomic and proteomic data indicate that MSTN inactivation could promote survival and resistance to apoptosis in muscle through the PI3K/Akt/GSK-3β survival pathway and through a mechanism that involves regulation of pro- and anti-apoptotic factors. A combination of increased potential for PI3K activation and decreased myonuclear apoptosis could be crucial to muscle hypertrophy in *MSTN*-null mice (Figure 2).

## Methods

### Muscle samples

The *MSTN*-null mice have a constitutive invalidation of *myostatin *gene [7]. These mice were bred at the Iowa State University, were slaughtered at 5 weeks postnatally, were dissected and their muscle masses were weighted. At this age the mice were nearly adults and this developmental stage was chosen in order to dissociate the influence of MSTN on hypertrophy from that of myofiber hyperplasia. Muscle samples were taken from *Quadriceps *muscle (mixed slow oxidative) immediately after slaughter, frozen in liquid nitrogen and kept at -80°C until protein extraction was performed. The *Quadriceps *muscle was chosen for the genomic experiments owing to its weight and marked hypertrophy (+87%, data not shown).

### Microarray experiments

Transcriptomic analysis was performed with a microarray of 6,473 genes expressed in muscle ("Myochips" available from OUEST-genopole^®^, France) as described in [28]. These Myochips consisted in 6,473 oligonucleotides from human (76%) and murine (24%) origin and 919 controls. Microarray experiments were performed according to recently proposed standards (MIAME consortium). Data were incorporated into the BASE database and the NCBI Gene Expression Omnibus (GEO)  and are accessible through GEO Series GSE5561 and GSE5456.

Total RNA was extracted from muscle tissue samples with TRIZOL^® ^reagent (Life Technologies) according to the manufacturer's recommendation. The RNA was then purified and treated with DNAse using the RNeasy^® ^Mini kit (Qiagen, France). RNA integrity was checked using Lab Chip Agilent technology, as previously described [28]. Each individual sample was compared to a reference pool consisting of skeletal muscle transcripts isolated from different muscles of four mice lines (Balb, C57bl6, B129) of different age and gender, and from cultured murine myogenic cells (C2C12).

Labeling was performed using a "Pronto Plus Direct Systems" kit (Corning-Promega) according to the supplier's guidelines. Five μg of total RNA was reverse transcribed into cDNA in the presence of cyanine (Cy3-dCTP/Cy5-dCTP, Amersham). cDNA purification was performed according to the manufacturer's instructions, and concentration and frequency of incorporation were determined by spectrophotometry. Three chips were hybridized per sample comparison. After washing, the chips were scanned on an Affymetrix 428™ Array Scanner. Images were analyzed using GenePix Pro V6 software (Axon instrument, Inc). Raw intensity data were normalized using the MADSCAN program (, 15). In order to identify differentially expressed genes, the Cy3/Cy5 ratios were statistically analyzed using Significance Analysis of Microarrays (SAM) method (FDR <5%; [64]). With the available sample size (4 animals per group) and 3 repeated measurements per sample and taking account a test power (1-β) set at 80% [65,66] and the biological variability (Standard Deviation SD = 0.083), the minimum difference was 11% corresponding to a threshold fold-change (FC) of 1.11. Thus we only retained appropriately powered genes.

### Proteomics

#### Preparation of muscle samples for 2-DE

Sample preparation was performed according to Bouley *et al*. [67]. Briefly, frozen muscle tissue (50–100 mg) was homogenized in a lysis buffer containing 8.3 M urea, 2 M thiourea, 1% DTT, 2% CHAPS and 2% IPG buffer pH 3–10 with a polytron and centrifuged at 10,000 *g *for 30 min. The supernatant was harvested and protein concentration for each sample was determined as described in the PlusOne 2-DE Quant kit (Amersham Biosciences).

Protein quantification was determined using the 2-DE Quant kit (Amersham Uppsala, Sweden), with bovine serum albumin as a standard. Average amount of protein was 9.6 μg. μl^-1 ^± 1.48 μg.μl^-1^.

#### 2-DE separation

2-DE separation was performed according to the method of Bouley *et al*. [67]. Briefly, for the first dimension (IEF), proteins were solubilized in a rehydration solution (8 M urea, 1 M thiourea, 0.28% DTT, 2% CHAPS, 2% IPG buffer pH 4–7 and 0.01% CBBR-250). IPG strips, 18 cm, covering a pH range of 4–7 were rehydrated overnight in 330 ml of this protein solution under low viscosity paraffin oil (sample loading by rehydration). IPG strips containing 700 μg of proteins were subjected to IEF (73.5 kVh) in a Multiphor II gel apparatus at 20.5°C. After completion of the IEF, proteins on the strip were equilibrated for 15 min in a buffer containing 6 M urea, 1% DTT, 30% glycerol, 50 mM Tris base, 2% sodium dodecyl sulphate (SDS), and DTT and then for an additional 15 min in the same solution except that DTT was replaced by 5% iodoacetamide and 0.002% bromophenol blue was added. The IPG strips were then transferred onto 11% T, 2.6% C separating polyacrylamide gels, and proteins were separated in the second dimension using a Hoefer DALTsix system (Hoefer, Scientific Instruments, San Francisco, CA, USA). 2-DE gels were stained using a colloidal CBB G-250 procedure [67] and digitized on a calibrated scanner (ImageScanner, Amersham).

#### Image and Data analysis

ImageMaster 2D Platinum software (Amersham Biosciences) was used to match and quantify protein spots on 2-DE gels. Parameters used for spot detection were minimal area = 70 pixels, smooth factor = 2.0, and saliency = 20. An artificial reference gel was created combining all the spots present in the different gels. The reference gel was then used for matching of corresponding protein spots between gels. The volume of individual spot was normalized (% volume) according to the mean volume of 249 protein spots which were strictly common to all gels. Comparisons between groups were performed using SAM statistical test [68]. Differences were declared significant for FDR <5%. Taking into account the biological variability which is 21%, the power analysis [66] allowed retaining proteins which present a minimum difference of 41% corresponding to a threshold FC of 1.41.

#### Identification of spots by Mass Spectrometry

All differential protein spots were excised from the polyacrylamide gel and placed in a Multiscreen solvinert 96-well filtration system plate (Millipore Corp., Bedford, MA). These proteins of interest were digested proteolytically in the plate with 3 volumes of trypsin solution (10 ng/μL; V5111, Promega, Madison, WI) at 37°C for 5 h and extracted with 8–12 μL of ACN. The resulting peptide fragments were mixed with an equal volume of CHCA saturated solution in 0.1% TFA, 50% aqueous ACN.

Peptide mass fingerprintings (PMF) of tryptic peptides were identified in a positive reflectron mode using a voyager DE Pro MALDI-TOF (Perspective Biosystem, Farminghan, MA, USA). External calibration was performed with a standard peptide solution (Proteomix, LaserBio Labs, Sophia-Antipolis, France). Internal calibration was performed using peptides resulting from auto-digestion of porcine trypsin, with protonated masses of 842.509, 1045.564, and 2211.104 Da. Peptides mass fingerprints were compared with mammalian databases [NCBI  and SWISS-PROT ] using MASCOT and ProFound software [ and , respectively (June 2007 and October 2007)].

The search criteria used were 1 missing trypsin cleavage site, partial methionine oxidation, partial carbamidomethylation of cysteine and mass deviation lower than 30 ppm. We required at least 5 matched peptides per protein for identification and used the ProFound and MASCOT probabilistic score and the accuracy of the experimental-to-theoretical isoelectric point (pI) and molecular weight (Mr).

#### Bioinformatic tools

To understand biological function or molecular mechanism of the main molecular targets of MSTN, we used a set of bioinformatics web tools such as Genomatix, DAVID and PANTHER for gene identification and String for interactions networks between proteins. The data mining allowed to visualize KEGG (Kyoto Encyclopedia of Genes and Genomes) pathway and to classify gene and protein by their function.

### Validation of Proteins and genes expression

#### Western blotting

Western-blotting was used to validate the main differentially expressed proteins. These proteins were separated by gel electrophoresis using SDS-PAGE for 2 h. After migration, the proteins were transferred onto PVDF transfer membrane Millipore (Dutscher Dominique SAS, France). Membranes were then blocked with 5% non-fat milk in T-TBS 1× buffer containing (blocking solution) and incubated under gentle agitation all the night at room temperature in the presence of the a primary antibodies: DJ-1, 1:2000 dilution of purified rabbit polyclonal anti-DJ-1 antibody (sc 32874, Santa Cruz); MyBP, 1:4000 dilution of purified mouse monoclonal anti-MyBP antibody (H0000 4608-MO1, ABNOVA); TCTP, 1:2000 dilution of purified rabbit polyclonal anti-HRF antibody (sc 30124, Santa Cruz); 14-3-3ε, 1:4000 dilution of purified goat polyclonal anti-14-3-3ε protein antibody (sc 1615, Santa Cruz); α-1 syntrophin, 1:500 dilution of purified rabbit polyclonal anti-SNTA1 antibody (sc- 50460, Santa Cruz); H-FABP, 1:2000 dilution of purified rabbit polyclonal anti-H-FABP, was a gift from Dr. Jacques Veerkamp and Dr. Herman Van Moerkerk (Dept. Biochemistry, Nijmegen, Netherlands) [63]; mortalin, 1:250 dilution of purified mouse monoclonal anti-GRP75 antibody (MAB3584, Clone 419612, R&D systems); PINK1, 1:2000 dilution of purified rabbit polyclonal anti-PINK1 antibody (sc- 33796, Santa Cruz); GSK-3β, 1:2000 dilution of purified rabbit polyclonal anti-GSK-3β antibody (sc- 9166, Santa Cruz); GSK-3β (ser9), 1:4000 dilution of purified goat polyclonal anti-p-GSK-3β (Ser9) antibody (sc- 11757, Santa Cruz); MyHC, 1:2000 dilution of purified mouse monoclonal anti-MyHC antibody [S5 8H2, lot: 9013-1, obtained under "Noé" project who associates our team and Biocytex company [69] and now this antibody is commercialized by Agro-Bio society]; PTEN, 1:2000 dilution of purified rabbit polyclonal anti-PTEN antibody (sc- 9145, Santa Cruz); p-Akt, 1:2000 dilution of purified rabbit polyclonal anti-p-Akt 1/2/3 (Ser473) antibody (sc- 33437-R, Santa Cruz); Actin, 1:20000 dilution of purified mouse monoclonal anti-actin antibody (IgG2a, Clone AC40, Sigma Chemical Company, St Louis, MO, USA), which are able to bind to its specific protein. The blots were extensively washed with Tris buffer and incubated under gentle agitation with the secondary antibodies (anti-mouse, anti-goat or anti-rabbit IgG combined with conjugated with horseradish peroxidase 1: 20000 dilution) for 1 h. The activity of the enzyme was revealed by Enhanced Chemiluminescence kit (Amersham).

The power analysis [66] allowed retaining proteins which present a minimum difference of 45% corresponding to a threshold FC of 1.45.

#### Real-Time Polymerase Chain Reaction Analysis

Seven genes shown to be differentially expressed were selected from the results obtained by microarray analysis and by proteomic analysis. The quantitative RT-PCR (qPCR) was realized using SYBR Green I dye. PCRs were performed using gene-specific primers pairs (Table 4). The absolute quantity of each target gene was determined by using serially diluted standards to generate a standard curve. The concentration in pg/μmol of each gene was determined by using a linear relationship between amounts of standards cDNA and their Ct (Cycle threshold). Real-Time PCR experiment was controlled using the ribosomal protein S6 as reference gene. The power analysis [66] allowed retaining genes which present a minimum difference of 54% corresponding to a threshold FC of 1.54.

**Table 4:** Primer sequences used in quantitative real-time PCR.

Gene Symbol	Forward primer	Reverse primer
DJ-1	AACACACCCACTGGCTAAGG	GGGCTTGGGCTCTAGTCTTT
Ywhae	GAGGTGTTTTGGGGGAGTTT	GGCTTCCATACCACCTTCAA
HSPA9	GCCGTTTCCAGTGCAACAAG	GATGCTGCCGTCCTGATGTT
GSK-3β	CTCTGGCCACCATCCTTATC	GTTCAGGTGGAGTTGGAAGC
PTEN	TGCAGAAAGACTTGAAGGTG	ATAAGTTCTAGCTGTGGTGG
Pi3Kr3	ATGAGAACCTGCCGCATTAT	GAATGCACCATCTGGTTTCC
mfgf6	ATTGGGAAAGCGGCTATTTGC	CTCGTGTGTTCCACTGATGC
mS6R	TTTGATTCTGAAAGCCATGCG	CGGTCCATCAGGATTCTATTG

All primer sequences were designed with the Primer3 software.

DJ-1: cancer- and Parkinson's disease-associated protein (park7); 14-3-3E: 14-3-3ε protein; HSPA9: mortalin, GSK-3β: Glycogen synthase kinase 3β; PI3KR3: Phosphatidylinositol 3-kinase regulatory subunit; PTEN: tumour-suppressor phosphatase with tensin homology; mfgf6: murine Fibroblast growth factor 6; mS6R: mouse ribosomal protein S6.

#### Western-blot and RT-PCR data analysis

The difference of proteins and genes levels between myostatin-null mice and their controls was analyzed by Student t-test. Results are expressed as the mean ± standard error of mean (SEM). A difference between the two groups was considered significant when *P *< 0.05.

## Abbreviations

MSTN: myostatin; SAM: Significance Analysis of Microarrays; DNA: Desoxyribo Nucleic Acid; FC: Fold Change; FDR: False Discovery Rate; PI3K: the Phosphatidyl Inositol 3-kinase.

## Authors' contributions

IC and ICM carried out the experiments. ICM conceived the experimental design in collaboration with BP and JFH who provided useful advice for data analysis and interpretation. IC and BM were actively involved in data analyses and interpretation. JR bred the experimental animals and provided muscle samples. CC provided the array and skilled advice for microarray analyses. IC and ICM wrote the manuscript. All authors read the manuscript, significantly contributed either to the presentation, the interpretation or the discussion of the results, and were highly involved in writing the manuscript.

## Supplementary Material

Additional file 1 **Up-regulated gene list with fold change (FC)**. The data provided here represent the statistical analysis (SAM) of up-regulated genes in the muscles of *MSTN-null *mice. Click here for file

Additional file 2 **Down-regulated gene list with fold change (FC)**. The data provided here represent the statistical analysis (SAM) of down-regulated genes in the muscles of *MSTN-null *mice. Click here for file
