# Supplementary material for: Molecular profiles of Quadriceps muscle in myostatin-null mice reveal PI3K and apoptotic pathways as myostatin targets
Source: BMC Genomics. 2009 Apr 27;10:196. doi: 10.1186/1471-2164-10-196 (PMC2684550; doi:10.1186/1471-2164-10-196)
Supplement: Additional file 1 — Up-regulated gene list with fold change (FC). The data provided here represent the statistical analysis (SAM) of up-regulated genes in the muscles of MSTN-null mice. [file 1471-2164-10-196-S1.doc]

**Additional file 1.**

**Up-regulated gene list with fold change (FC).**

| [**Gene Name**](http://genome-www4.stanford.edu/cgi-bin/SMD/source/sourceResult?choice=Gene&option=Name&criteria=DTYMK__________-1A) | **FC** | [**Gene Name**](http://genome-www4.stanford.edu/cgi-bin/SMD/source/sourceResult?choice=Gene&option=Name&criteria=DTYMK__________-1A) | **FC** | [**Gene Name**](http://genome-www4.stanford.edu/cgi-bin/SMD/source/sourceResult?choice=Gene&option=Name&criteria=DTYMK__________-1A) | **FC** | [**Gene Name**](http://genome-www4.stanford.edu/cgi-bin/SMD/source/sourceResult?choice=Gene&option=Name&criteria=DTYMK__________-1A) | **FC** |
| --- | --- | --- | --- | --- | --- | --- | --- |
| [ANXA6](http://genome-www4.stanford.edu/cgi-bin/SMD/source/sourceResult?choice=Gene&option=Name&criteria=9330197E15Rik__-1A) | 1.30 | [Alk](http://genome-www4.stanford.edu/cgi-bin/SMD/source/sourceResult?choice=Gene&option=Name&criteria=AMPD3__________-1A) | 1.10 | [DAD1](http://genome-www4.stanford.edu/cgi-bin/SMD/source/sourceResult?choice=Gene&option=Name&criteria=M11S1__________-1A) | 1.10 | [MGC2793](http://genome-www4.stanford.edu/cgi-bin/SMD/source/sourceResult?choice=Gene&option=Name&criteria=HGAK026713_____-1A) | 1.10 |
| [ITPK1](http://genome-www4.stanford.edu/cgi-bin/SMD/source/sourceResult?choice=Gene&option=Name&criteria=Pdcd6__________-1A) | 1.30 | [AMPD3](http://genome-www4.stanford.edu/cgi-bin/SMD/source/sourceResult?choice=Gene&option=Name&criteria=RAN____________-1A) | 1.10 | [Disp](http://genome-www4.stanford.edu/cgi-bin/SMD/source/sourceResult?choice=Gene&option=Name&criteria=RTN4___________-1A) | 1.10 | [MGC2817](http://genome-www4.stanford.edu/cgi-bin/SMD/source/sourceResult?choice=Gene&option=Name&criteria=G6PD___________-2A) | 1.10 |
| [1700026M20Rik](http://genome-www4.stanford.edu/cgi-bin/SMD/source/sourceResult?choice=Gene&option=Name&criteria=PDF____________-1A) | 1.20 | [Ang](http://genome-www4.stanford.edu/cgi-bin/SMD/source/sourceResult?choice=Gene&option=Name&criteria=FAM13C1________-1A) | 1.10 | [DMBT1](http://genome-www4.stanford.edu/cgi-bin/SMD/source/sourceResult?choice=Gene&option=Name&criteria=Ppox___________-1A) | 1.10 | [MINK](http://genome-www4.stanford.edu/cgi-bin/SMD/source/sourceResult?choice=Gene&option=Name&criteria=FLJ20574_______-1A) | 1.10 |
| [2610312B22Rik](http://genome-www4.stanford.edu/cgi-bin/SMD/source/sourceResult?choice=Gene&option=Name&criteria=Dpf3___________-1A) | 1.20 | [Atp6v0a4](http://genome-www4.stanford.edu/cgi-bin/SMD/source/sourceResult?choice=Gene&option=Name&criteria=Psmc5__________-1A) | 1.10 | [Dpf3](http://genome-www4.stanford.edu/cgi-bin/SMD/source/sourceResult?choice=Gene&option=Name&criteria=1700026M20Rik__-1A) | 1.10 | [MO25](http://genome-www4.stanford.edu/cgi-bin/SMD/source/sourceResult?choice=Gene&option=Name&criteria=2810036M19Rik__-1A) | 1.10 |
| [5830475F03Rik](http://genome-www4.stanford.edu/cgi-bin/SMD/source/sourceResult?choice=Gene&option=Name&criteria=SALPR__________-1A) | 1.20 | [ATP6V1F](http://genome-www4.stanford.edu/cgi-bin/SMD/source/sourceResult?choice=Gene&option=Name&criteria=ADH1B__________-1A) | 1.10 | [DTYMK](http://genome-www4.stanford.edu/cgi-bin/SMD/source/sourceResult?choice=Gene&option=Name&criteria=Abcd3__________-1A) | 1.10 | [Mre11a](http://genome-www4.stanford.edu/cgi-bin/SMD/source/sourceResult?choice=Gene&option=Name&criteria=MGC2793________-1A) | 1.10 |
| [AARS](http://genome-www4.stanford.edu/cgi-bin/SMD/source/sourceResult?choice=Gene&option=Name&criteria=Csnk2a1________-1A) | 1.20 | [Atpi](http://genome-www4.stanford.edu/cgi-bin/SMD/source/sourceResult?choice=Gene&option=Name&criteria=Atp6v0a4_______-1A) | 1.10 | [Eef1b2](http://genome-www4.stanford.edu/cgi-bin/SMD/source/sourceResult?choice=Gene&option=Name&criteria=C11ORF10_______-1A) | 1.10 | [MRPL15](http://genome-www4.stanford.edu/cgi-bin/SMD/source/sourceResult?choice=Gene&option=Name&criteria=H2BFS__________-1A) | 1.10 |
| [Abcd3](http://genome-www4.stanford.edu/cgi-bin/SMD/source/sourceResult?choice=Gene&option=Name&criteria=ELN____________-1A) | 1.20 | [B:0792](http://genome-www4.stanford.edu/cgi-bin/SMD/source/sourceResult?choice=Gene&option=Name&criteria=LOC162427______-1A) | 1.10 | [EFNA4](http://genome-www4.stanford.edu/cgi-bin/SMD/source/sourceResult?choice=Gene&option=Name&criteria=Phxr2__________-1A) | 1.10 | MYL1 | 1.10 |
| [Actb](http://genome-www4.stanford.edu/cgi-bin/SMD/source/sourceResult?choice=Gene&option=Name&criteria=Ppp1cb_________-1A) | 1.20 | [B:7699](http://genome-www4.stanford.edu/cgi-bin/SMD/source/sourceResult?choice=Gene&option=Name&criteria=Grm8___________-1A) | 1.10 | [Ehd3](http://genome-www4.stanford.edu/cgi-bin/SMD/source/sourceResult?choice=Gene&option=Name&criteria=Actg2__________-1A) | 1.10 | [Npm1](http://genome-www4.stanford.edu/cgi-bin/SMD/source/sourceResult?choice=Gene&option=Name&criteria=PEX11B_________-1A) | 1.10 |
| [Alas1](http://genome-www4.stanford.edu/cgi-bin/SMD/source/sourceResult?choice=Gene&option=Name&criteria=FAM14A_________-1A) | 1.20 | [B4galt6](http://genome-www4.stanford.edu/cgi-bin/SMD/source/sourceResult?choice=Gene&option=Name&criteria=P4HB___________-1A) | 1.10 | [EIF3S10](http://genome-www4.stanford.edu/cgi-bin/SMD/source/sourceResult?choice=Gene&option=Name&criteria=PTPRF__________-1A) | 1.10 | [Oprs1](http://genome-www4.stanford.edu/cgi-bin/SMD/source/sourceResult?choice=Gene&option=Name&criteria=2310047C04Rik__-1A) | 1.10 |
| [BCL7A](http://genome-www4.stanford.edu/cgi-bin/SMD/source/sourceResult?choice=Gene&option=Name&criteria=AARS___________-1A) | 1.20 | [Banp](http://genome-www4.stanford.edu/cgi-bin/SMD/source/sourceResult?choice=Gene&option=Name&criteria=Fancl__________-1A) | 1.10 | [ELN](http://genome-www4.stanford.edu/cgi-bin/SMD/source/sourceResult?choice=Gene&option=Name&criteria=FLJ11151_______-1A) | 1.10 | [Ovgp1](http://genome-www4.stanford.edu/cgi-bin/SMD/source/sourceResult?choice=Gene&option=Name&criteria=C14ORF140______-1A) | 1.10 |
| [C:7761](http://genome-www4.stanford.edu/cgi-bin/SMD/source/sourceResult?choice=Gene&option=Name&criteria=MEF2C__________-1A) | 1.20 | BIRC5 | 1.10 | [FAM13C1](http://genome-www4.stanford.edu/cgi-bin/SMD/source/sourceResult?choice=Gene&option=Name&criteria=GMFG___________-1A) | 1.10 | [P4HB](http://genome-www4.stanford.edu/cgi-bin/SMD/source/sourceResult?choice=Gene&option=Name&criteria=Ndufa10________-1A) | 1.10 |
| CLONEIGA5135IMM | 1.20 | [Btnl4](http://genome-www4.stanford.edu/cgi-bin/SMD/source/sourceResult?choice=Gene&option=Name&criteria=C10orf3________-1A) | 1.10 | [Fancl](http://genome-www4.stanford.edu/cgi-bin/SMD/source/sourceResult?choice=Gene&option=Name&criteria=Reck___________-1A) | 1.10 | [PDE5A](http://genome-www4.stanford.edu/cgi-bin/SMD/source/sourceResult?choice=Gene&option=Name&criteria=Pecam__________-1A) | 1.10 |
| [CPD](http://genome-www4.stanford.edu/cgi-bin/SMD/source/sourceResult?choice=Gene&option=Name&criteria=Mre11a_________-1A) | 1.20 | [C:1860](http://genome-www4.stanford.edu/cgi-bin/SMD/source/sourceResult?choice=Gene&option=Name&criteria=Copz1__________-1A) | 1.10 | [FBLN5](http://genome-www4.stanford.edu/cgi-bin/SMD/source/sourceResult?choice=Gene&option=Name&criteria=Foxa3__________-1A) | 1.10 | [PDE6B](http://genome-www4.stanford.edu/cgi-bin/SMD/source/sourceResult?choice=Gene&option=Name&criteria=C:1947_________-1A) | 1.10 |
| [D630041K24Rik](http://genome-www4.stanford.edu/cgi-bin/SMD/source/sourceResult?choice=Gene&option=Name&criteria=CKAP4__________-1A) | 1.20 | [C:1947](http://genome-www4.stanford.edu/cgi-bin/SMD/source/sourceResult?choice=Gene&option=Name&criteria=DAD1___________-1A) | 1.10 | [FLJ10901](http://genome-www4.stanford.edu/cgi-bin/SMD/source/sourceResult?choice=Gene&option=Name&criteria=FUBP3__________-1A) | 1.10 | [PDF](http://genome-www4.stanford.edu/cgi-bin/SMD/source/sourceResult?choice=Gene&option=Name&criteria=MO25___________-1A) | 1.10 |
| [DKFZP564K1964](http://genome-www4.stanford.edu/cgi-bin/SMD/source/sourceResult?choice=Gene&option=Name&criteria=Ppp4c__________-1A) | 1.20 | [C:2111](http://genome-www4.stanford.edu/cgi-bin/SMD/source/sourceResult?choice=Gene&option=Name&criteria=C:3400_________-1A) | 1.10 | [FLJ10956](http://genome-www4.stanford.edu/cgi-bin/SMD/source/sourceResult?choice=Gene&option=Name&criteria=1600015H20Rik__-1A) | 1.10 | [Pdha1](http://genome-www4.stanford.edu/cgi-bin/SMD/source/sourceResult?choice=Gene&option=Name&criteria=1700019B01Rik__-1A) | 1.10 |
| ENSG00000029271 | 1.20 | [C:3400](http://genome-www4.stanford.edu/cgi-bin/SMD/source/sourceResult?choice=Gene&option=Name&criteria=Hebp1__________-1A) | 1.10 | [FLJ11151](http://genome-www4.stanford.edu/cgi-bin/SMD/source/sourceResult?choice=Gene&option=Name&criteria=CCND3__________-1A) | 1.10 | [Pecam](http://genome-www4.stanford.edu/cgi-bin/SMD/source/sourceResult?choice=Gene&option=Name&criteria=RNF29__________-1A) | 1.10 |
| [FAM14A](http://genome-www4.stanford.edu/cgi-bin/SMD/source/sourceResult?choice=Gene&option=Name&criteria=ENSG00000029271-1A) | 1.20 | [C:3700](http://genome-www4.stanford.edu/cgi-bin/SMD/source/sourceResult?choice=Gene&option=Name&criteria=PRO2958________-1A) | 1.10 | [FLJ12132](http://genome-www4.stanford.edu/cgi-bin/SMD/source/sourceResult?choice=Gene&option=Name&criteria=CTSL___________-1A) | 1.10 | [PEX11B](http://genome-www4.stanford.edu/cgi-bin/SMD/source/sourceResult?choice=Gene&option=Name&criteria=C:3700_________-1A) | 1.10 |
| [FGF6](http://genome-www4.stanford.edu/cgi-bin/SMD/source/sourceResult?choice=Gene&option=Name&criteria=Col4a4_________-1A) | 1.20 | [C:3969](http://genome-www4.stanford.edu/cgi-bin/SMD/source/sourceResult?choice=Gene&option=Name&criteria=PDE5A__________-1A) | 1.10 | [FLJ20356](http://genome-www4.stanford.edu/cgi-bin/SMD/source/sourceResult?choice=Gene&option=Name&criteria=B:0792_________-1A) | 1.10 | [PHIP](http://genome-www4.stanford.edu/cgi-bin/SMD/source/sourceResult?choice=Gene&option=Name&criteria=A2BP1__________-1A) | 1.10 |
| [GALR2](http://genome-www4.stanford.edu/cgi-bin/SMD/source/sourceResult?choice=Gene&option=Name&criteria=FLJ10956_______-1A) | 1.20 | [C:5888](http://genome-www4.stanford.edu/cgi-bin/SMD/source/sourceResult?choice=Gene&option=Name&criteria=4833424P18Rik__-1A) | 1.10 | [FLJ20574](http://genome-www4.stanford.edu/cgi-bin/SMD/source/sourceResult?choice=Gene&option=Name&criteria=4932432N11Rik__-1A) | 1.10 | [PHOX2B](http://genome-www4.stanford.edu/cgi-bin/SMD/source/sourceResult?choice=Gene&option=Name&criteria=AGER___________-1A) | 1.10 |
| [KCNC4](http://genome-www4.stanford.edu/cgi-bin/SMD/source/sourceResult?choice=Gene&option=Name&criteria=Odz3___________-1A) | 1.20 | [C:7436](http://genome-www4.stanford.edu/cgi-bin/SMD/source/sourceResult?choice=Gene&option=Name&criteria=CHAF1B_________-1A) | 1.10 | [Foxa3](http://genome-www4.stanford.edu/cgi-bin/SMD/source/sourceResult?choice=Gene&option=Name&criteria=FLJ10901_______-1A) | 1.10 | [Phxr2](http://genome-www4.stanford.edu/cgi-bin/SMD/source/sourceResult?choice=Gene&option=Name&criteria=PHIP___________-1A) | 1.10 |
| [LTB4R](http://genome-www4.stanford.edu/cgi-bin/SMD/source/sourceResult?choice=Gene&option=Name&criteria=GALR2__________-1A) | 1.20 | [C:7950](http://genome-www4.stanford.edu/cgi-bin/SMD/source/sourceResult?choice=Gene&option=Name&criteria=FBLN5__________-1A) | 1.10 | [FUBP3](http://genome-www4.stanford.edu/cgi-bin/SMD/source/sourceResult?choice=Gene&option=Name&criteria=CCL22__________-1A) | 1.10 | [PIK3CG](http://genome-www4.stanford.edu/cgi-bin/SMD/source/sourceResult?choice=Gene&option=Name&criteria=PLCG1__________-1A) | 1.10 |
| [MEF2C](http://genome-www4.stanford.edu/cgi-bin/SMD/source/sourceResult?choice=Gene&option=Name&criteria=MAP2K7_________-1A) | 1.20 | [C:8496](http://genome-www4.stanford.edu/cgi-bin/SMD/source/sourceResult?choice=Gene&option=Name&criteria=Kng____________-1A) | 1.10 | [G6PD](http://genome-www4.stanford.edu/cgi-bin/SMD/source/sourceResult?choice=Gene&option=Name&criteria=Banp___________-1A) | 1.10 | [PIK3R3](http://genome-www4.stanford.edu/cgi-bin/SMD/source/sourceResult?choice=Gene&option=Name&criteria=LOC51039_______-1A) | 1.10 |
| [Nr4a2](http://genome-www4.stanford.edu/cgi-bin/SMD/source/sourceResult?choice=Gene&option=Name&criteria=Hp_____________-1A) | 1.20 | [C:8792](http://genome-www4.stanford.edu/cgi-bin/SMD/source/sourceResult?choice=Gene&option=Name&criteria=KIAA1430_______-2A) | 1.10 | [Gabrq](http://genome-www4.stanford.edu/cgi-bin/SMD/source/sourceResult?choice=Gene&option=Name&criteria=Ptpn12_________-1A) | 1.10 | [PINK1](http://genome-www4.stanford.edu/cgi-bin/SMD/source/sourceResult?choice=Gene&option=Name&criteria=MurinemRNAforT--1A) | 1.10 |
| [Odz3](http://genome-www4.stanford.edu/cgi-bin/SMD/source/sourceResult?choice=Gene&option=Name&criteria=Ang____________-1A) | 1.20 | [C10orf3](http://genome-www4.stanford.edu/cgi-bin/SMD/source/sourceResult?choice=Gene&option=Name&criteria=FLJ12547_______-1A) | 1.10 | [Gas1](http://genome-www4.stanford.edu/cgi-bin/SMD/source/sourceResult?choice=Gene&option=Name&criteria=MGC2817________-1A) | 1.10 | [PLA2G6](http://genome-www4.stanford.edu/cgi-bin/SMD/source/sourceResult?choice=Gene&option=Name&criteria=LOC150580______-1A) | 1.10 |
| [Pdcd6](http://genome-www4.stanford.edu/cgi-bin/SMD/source/sourceResult?choice=Gene&option=Name&criteria=SEMA3A_________-1A) | 1.20 | [C11ORF10](http://genome-www4.stanford.edu/cgi-bin/SMD/source/sourceResult?choice=Gene&option=Name&criteria=DKFZP564K1964__-1A) | 1.10 | [Gclc](http://genome-www4.stanford.edu/cgi-bin/SMD/source/sourceResult?choice=Gene&option=Name&criteria=KCNJ15_________-1A) | 1.10 | [PLCG1](http://genome-www4.stanford.edu/cgi-bin/SMD/source/sourceResult?choice=Gene&option=Name&criteria=PRO2831________-1A) | 1.10 |
| [Pdlim1](http://genome-www4.stanford.edu/cgi-bin/SMD/source/sourceResult?choice=Gene&option=Name&criteria=CTSS___________-1A) | 1.20 | [C14ORF140](http://genome-www4.stanford.edu/cgi-bin/SMD/source/sourceResult?choice=Gene&option=Name&criteria=Btnl4__________-1A) | 1.10 | [GMFG](http://genome-www4.stanford.edu/cgi-bin/SMD/source/sourceResult?choice=Gene&option=Name&criteria=EFNA4__________-1A) | 1.10 | [Pmp22](http://genome-www4.stanford.edu/cgi-bin/SMD/source/sourceResult?choice=Gene&option=Name&criteria=C:8792_________-1A) | 1.10 |
| [PNUTL2](http://genome-www4.stanford.edu/cgi-bin/SMD/source/sourceResult?choice=Gene&option=Name&criteria=Alas1__________-1A) | 1.20 | [C14ORF91](http://genome-www4.stanford.edu/cgi-bin/SMD/source/sourceResult?choice=Gene&option=Name&criteria=Nr4a2__________-1A) | 1.10 | [GNRPX](http://genome-www4.stanford.edu/cgi-bin/SMD/source/sourceResult?choice=Gene&option=Name&criteria=Aldh3a2________-1A) | 1.10 | [Ppox](http://genome-www4.stanford.edu/cgi-bin/SMD/source/sourceResult?choice=Gene&option=Name&criteria=MINK___________-1A) | 1.10 |
| [Postn](http://genome-www4.stanford.edu/cgi-bin/SMD/source/sourceResult?choice=Gene&option=Name&criteria=Npm1___________-1A) | 1.20 | [CCL20](http://genome-www4.stanford.edu/cgi-bin/SMD/source/sourceResult?choice=Gene&option=Name&criteria=2410116I05Rik__-1A) | 1.10 | [Gpr39](http://genome-www4.stanford.edu/cgi-bin/SMD/source/sourceResult?choice=Gene&option=Name&criteria=RPL18__________-1A) | 1.10 | [Ppp4c](http://genome-www4.stanford.edu/cgi-bin/SMD/source/sourceResult?choice=Gene&option=Name&criteria=C:7436_________-1A) | 1.10 |
| [Ppp1cb](http://genome-www4.stanford.edu/cgi-bin/SMD/source/sourceResult?choice=Gene&option=Name&criteria=Alk____________-1A) | 1.20 | [CCL22](http://genome-www4.stanford.edu/cgi-bin/SMD/source/sourceResult?choice=Gene&option=Name&criteria=5830411K18Rik__-1A) | 1.10 | [Grm8](http://genome-www4.stanford.edu/cgi-bin/SMD/source/sourceResult?choice=Gene&option=Name&criteria=C14ORF91_______-1A) | 1.10 | [PRKAR1B](http://genome-www4.stanford.edu/cgi-bin/SMD/source/sourceResult?choice=Gene&option=Name&criteria=DMBT1__________-1A) | 1.10 |
| [PVALB](http://genome-www4.stanford.edu/cgi-bin/SMD/source/sourceResult?choice=Gene&option=Name&criteria=Eef1b2_________-1A) | 1.20 | [CCND3](http://genome-www4.stanford.edu/cgi-bin/SMD/source/sourceResult?choice=Gene&option=Name&criteria=PLA2G6_________-1A) | 1.10 | [GTF3C1](http://genome-www4.stanford.edu/cgi-bin/SMD/source/sourceResult?choice=Gene&option=Name&criteria=RPS17__________-1A) | 1.10 | [PRO2831](http://genome-www4.stanford.edu/cgi-bin/SMD/source/sourceResult?choice=Gene&option=Name&criteria=CTNS___________-1A) | 1.10 |
| [SEMA3A](http://genome-www4.stanford.edu/cgi-bin/SMD/source/sourceResult?choice=Gene&option=Name&criteria=PIK3CG_________-1A) | 1.20 | [CCR7](http://genome-www4.stanford.edu/cgi-bin/SMD/source/sourceResult?choice=Gene&option=Name&criteria=PDE6B__________-1A) | 1.10 | [GUK1](http://genome-www4.stanford.edu/cgi-bin/SMD/source/sourceResult?choice=Gene&option=Name&criteria=PRSS11_________-1A) | 1.10 | [PRO2958](http://genome-www4.stanford.edu/cgi-bin/SMD/source/sourceResult?choice=Gene&option=Name&criteria=Prps1__________-1A) | 1.10 |
| [1600015H20Rik](http://genome-www4.stanford.edu/cgi-bin/SMD/source/sourceResult?choice=Gene&option=Name&criteria=CLONE82T-CELLRE-1A) | 1.10 | [CD164](http://genome-www4.stanford.edu/cgi-bin/SMD/source/sourceResult?choice=Gene&option=Name&criteria=2410001H17Rik__-1A) | 1.10 | [Gyg1](http://genome-www4.stanford.edu/cgi-bin/SMD/source/sourceResult?choice=Gene&option=Name&criteria=Pdlim1_________-1A) | 1.10 | [Prps1](http://genome-www4.stanford.edu/cgi-bin/SMD/source/sourceResult?choice=Gene&option=Name&criteria=Cherp__________-1A) | 1.10 |
| [1700016D08Rik](http://genome-www4.stanford.edu/cgi-bin/SMD/source/sourceResult?choice=Gene&option=Name&criteria=CDH8___________-1A) | 1.10 | CDH8 | 1.10 | [H2BFS](http://genome-www4.stanford.edu/cgi-bin/SMD/source/sourceResult?choice=Gene&option=Name&criteria=CCL20__________-1A) | 1.10 | [PRSS11](http://genome-www4.stanford.edu/cgi-bin/SMD/source/sourceResult?choice=Gene&option=Name&criteria=4930428F12Rik__-1A) | 1.10 |
| [1700080G18Rik](http://genome-www4.stanford.edu/cgi-bin/SMD/source/sourceResult?choice=Gene&option=Name&criteria=MRPL15_________-1A) | 1.10 | [CERCAM](http://genome-www4.stanford.edu/cgi-bin/SMD/source/sourceResult?choice=Gene&option=Name&criteria=Gas1___________-1A) | 1.10 | [Hebp1](http://genome-www4.stanford.edu/cgi-bin/SMD/source/sourceResult?choice=Gene&option=Name&criteria=SFRS7__________-1A) | 1.10 | [Psmc5](http://genome-www4.stanford.edu/cgi-bin/SMD/source/sourceResult?choice=Gene&option=Name&criteria=Og9x___________-1A) | 1.10 |
| [2310047C04Rik](http://genome-www4.stanford.edu/cgi-bin/SMD/source/sourceResult?choice=Gene&option=Name&criteria=Mfn1___________-1A) | 1.10 | [Cfi](http://genome-www4.stanford.edu/cgi-bin/SMD/source/sourceResult?choice=Gene&option=Name&criteria=MYL1___________-2A) | 1.10 | [HGAK026713](http://genome-www4.stanford.edu/cgi-bin/SMD/source/sourceResult?choice=Gene&option=Name&criteria=GUK1___________-1A) | 1.10 | [Ptgfr](http://genome-www4.stanford.edu/cgi-bin/SMD/source/sourceResult?choice=Gene&option=Name&criteria=Gabrq__________-1A) | 1.10 |
| [2410001H17Rik](http://genome-www4.stanford.edu/cgi-bin/SMD/source/sourceResult?choice=Gene&option=Name&criteria=1700123D08Rik__-1A) | 1.10 | [CFL2](http://genome-www4.stanford.edu/cgi-bin/SMD/source/sourceResult?choice=Gene&option=Name&criteria=CPD____________-1A) | 1.10 | [Hp](http://genome-www4.stanford.edu/cgi-bin/SMD/source/sourceResult?choice=Gene&option=Name&criteria=C:3969_________-1A) | 1.10 | [PTPRF](http://genome-www4.stanford.edu/cgi-bin/SMD/source/sourceResult?choice=Gene&option=Name&criteria=MADH6__________-1A) | 1.10 |
| [2810036M19Rik](http://genome-www4.stanford.edu/cgi-bin/SMD/source/sourceResult?choice=Gene&option=Name&criteria=Inppl1_________-1A) | 1.10 | [CHAF1B](http://genome-www4.stanford.edu/cgi-bin/SMD/source/sourceResult?choice=Gene&option=Name&criteria=Atpi___________-1A) | 1.10 | [Inppl1](http://genome-www4.stanford.edu/cgi-bin/SMD/source/sourceResult?choice=Gene&option=Name&criteria=Gclc___________-1A) | 1.10 | [Rad50](http://genome-www4.stanford.edu/cgi-bin/SMD/source/sourceResult?choice=Gene&option=Name&criteria=CD164__________-1A) | 1.10 |
| [4833424P18Rik](http://genome-www4.stanford.edu/cgi-bin/SMD/source/sourceResult?choice=Gene&option=Name&criteria=C:1860_________-1A) | 1.10 | [Cherp](http://genome-www4.stanford.edu/cgi-bin/SMD/source/sourceResult?choice=Gene&option=Name&criteria=LOXL1__________-1A) | 1.10 | [KCNJ15](http://genome-www4.stanford.edu/cgi-bin/SMD/source/sourceResult?choice=Gene&option=Name&criteria=CXCR3__________-1A) | 1.10 | [RAN](http://genome-www4.stanford.edu/cgi-bin/SMD/source/sourceResult?choice=Gene&option=Name&criteria=Disp___________-1A) | 1.10 |
| 5830411K18Rik | 1.10 | [CKAP4](http://genome-www4.stanford.edu/cgi-bin/SMD/source/sourceResult?choice=Gene&option=Name&criteria=SLC6A6_________-1A) | 1.10 | [Khdrbs3](http://genome-www4.stanford.edu/cgi-bin/SMD/source/sourceResult?choice=Gene&option=Name&criteria=CERCAM_________-1A) | 1.10 | [Reck](http://genome-www4.stanford.edu/cgi-bin/SMD/source/sourceResult?choice=Gene&option=Name&criteria=PINK1__________-1A) | 1.10 |
| [6720473M11Rik](http://genome-www4.stanford.edu/cgi-bin/SMD/source/sourceResult?choice=Gene&option=Name&criteria=Gpr39__________-1A) | 1.10 | CLONE82T-CELLRE | 1.10 | KIAA1430 | 1.10 | [RLUCL](http://genome-www4.stanford.edu/cgi-bin/SMD/source/sourceResult?choice=Gene&option=Name&criteria=FLJ20356_______-1A) | 1.10 |
| [9330197E15Rik](http://genome-www4.stanford.edu/cgi-bin/SMD/source/sourceResult?choice=Gene&option=Name&criteria=C:5888_________-1A) | 1.10 | CLONEIGA5487IMM | 1.10 | [Kng](http://genome-www4.stanford.edu/cgi-bin/SMD/source/sourceResult?choice=Gene&option=Name&criteria=ORMDL2_________-1A) | 1.10 | RNF29 | 1.10 |
| [A2BP1](http://genome-www4.stanford.edu/cgi-bin/SMD/source/sourceResult?choice=Gene&option=Name&criteria=Ors18__________-1A) | 1.10 | [Col4a4](http://genome-www4.stanford.edu/cgi-bin/SMD/source/sourceResult?choice=Gene&option=Name&criteria=GTF3C1_________-1A) | 1.10 | [Limd1](http://genome-www4.stanford.edu/cgi-bin/SMD/source/sourceResult?choice=Gene&option=Name&criteria=Rad50__________-1A) | 1.10 | [RPL18](http://genome-www4.stanford.edu/cgi-bin/SMD/source/sourceResult?choice=Gene&option=Name&criteria=D630041K24Rik__-1A) | 1.10 |
| [Abl1](http://genome-www4.stanford.edu/cgi-bin/SMD/source/sourceResult?choice=Gene&option=Name&criteria=PRKAR1B________-1A) | 1.10 | [Copz1](http://genome-www4.stanford.edu/cgi-bin/SMD/source/sourceResult?choice=Gene&option=Name&criteria=GNRPX__________-1A) | 1.10 | [LOC150580](http://genome-www4.stanford.edu/cgi-bin/SMD/source/sourceResult?choice=Gene&option=Name&criteria=B4galt6________-1A) | 1.10 | [RPS17](http://genome-www4.stanford.edu/cgi-bin/SMD/source/sourceResult?choice=Gene&option=Name&criteria=ACTG1__________-1A) | 1.10 |
| [ACTG1](http://genome-www4.stanford.edu/cgi-bin/SMD/source/sourceResult?choice=Gene&option=Name&criteria=1700080G18Rik__-1A) | 1.10 | [Csnk2a1](http://genome-www4.stanford.edu/cgi-bin/SMD/source/sourceResult?choice=Gene&option=Name&criteria=Ehd3___________-1A) | 1.10 | [LOC51039](http://genome-www4.stanford.edu/cgi-bin/SMD/source/sourceResult?choice=Gene&option=Name&criteria=ATP6V1F________-1A) | 1.10 | [RTN4](http://genome-www4.stanford.edu/cgi-bin/SMD/source/sourceResult?choice=Gene&option=Name&criteria=Ptgfr__________-1A) | 1.10 |
| [Actg2](http://genome-www4.stanford.edu/cgi-bin/SMD/source/sourceResult?choice=Gene&option=Name&criteria=Limd1__________-1A) | 1.10 | [CTNS](http://genome-www4.stanford.edu/cgi-bin/SMD/source/sourceResult?choice=Gene&option=Name&criteria=S100A10________-1A) | 1.10 | [LOXL1](http://genome-www4.stanford.edu/cgi-bin/SMD/source/sourceResult?choice=Gene&option=Name&criteria=BIRC5__________-2A) | 1.10 | [S100A10](http://genome-www4.stanford.edu/cgi-bin/SMD/source/sourceResult?choice=Gene&option=Name&criteria=C:8496_________-1A) | 1.10 |
| [ADH1B](http://genome-www4.stanford.edu/cgi-bin/SMD/source/sourceResult?choice=Gene&option=Name&criteria=1700016D08Rik__-1A) | 1.10 | [Ctps2](http://genome-www4.stanford.edu/cgi-bin/SMD/source/sourceResult?choice=Gene&option=Name&criteria=CTSK___________-1A) | 1.10 | [LRP1](http://genome-www4.stanford.edu/cgi-bin/SMD/source/sourceResult?choice=Gene&option=Name&criteria=CLONEIGA5487IMM-1A) | 1.10 | [SALPR](http://genome-www4.stanford.edu/cgi-bin/SMD/source/sourceResult?choice=Gene&option=Name&criteria=C:7950_________-1A) | 1.10 |
| [AGER](http://genome-www4.stanford.edu/cgi-bin/SMD/source/sourceResult?choice=Gene&option=Name&criteria=Pdha1__________-1A) | 1.10 | [CTSL](http://genome-www4.stanford.edu/cgi-bin/SMD/source/sourceResult?choice=Gene&option=Name&criteria=CCR7___________-1A) | 1.10 | [MADH6](http://genome-www4.stanford.edu/cgi-bin/SMD/source/sourceResult?choice=Gene&option=Name&criteria=RLUCL__________-1A) | 1.10 | [SFRS7](http://genome-www4.stanford.edu/cgi-bin/SMD/source/sourceResult?choice=Gene&option=Name&criteria=Oprs1__________-1A) | 1.10 |
| [Aldh3a2](http://genome-www4.stanford.edu/cgi-bin/SMD/source/sourceResult?choice=Gene&option=Name&criteria=Ctps2__________-1A) | 1.10 | [CTSS](http://genome-www4.stanford.edu/cgi-bin/SMD/source/sourceResult?choice=Gene&option=Name&criteria=PIK3R3_________-1A) | 1.10 | [MAP2K7](http://genome-www4.stanford.edu/cgi-bin/SMD/source/sourceResult?choice=Gene&option=Name&criteria=C:2111_________-1A) | 1.10 | [SLC6A6](http://genome-www4.stanford.edu/cgi-bin/SMD/source/sourceResult?choice=Gene&option=Name&criteria=PHOX2B_________-1A) | 1.10 |
