# Supplementary material for: Molecular profiles of Quadriceps muscle in myostatin-null mice reveal PI3K and apoptotic pathways as myostatin targets
Source: BMC Genomics. 2009 Apr 27;10:196. doi: 10.1186/1471-2164-10-196 (PMC2684550; doi:10.1186/1471-2164-10-196)
Supplement: Additional file 2 — Down-regulated gene list with fold change (FC). The data provided here represent the statistical analysis (SAM) of down-regulated genes in the muscles of MSTN-null mice. [file 1471-2164-10-196-S2.doc]

**Additional file 2.**

**Down-regulated gene list with fold change (FC).**

| [**Gene Name**](http://genome-www4.stanford.edu/cgi-bin/SMD/source/sourceResult?choice=Gene&option=Name&criteria=Muscarolicytoch-1A) | **FC** | [**Gene Name**](http://genome-www4.stanford.edu/cgi-bin/SMD/source/sourceResult?choice=Gene&option=Name&criteria=Muscarolicytoch-1A) | **FC** | [**Gene Name**](http://genome-www4.stanford.edu/cgi-bin/SMD/source/sourceResult?choice=Gene&option=Name&criteria=Muscarolicytoch-1A) | **FC** | [**Gene Name**](http://genome-www4.stanford.edu/cgi-bin/SMD/source/sourceResult?choice=Gene&option=Name&criteria=Muscarolicytoch-1A) | **FC** |
| --- | --- | --- | --- | --- | --- | --- | --- |
| [PRKACG](http://genome-www4.stanford.edu/cgi-bin/SMD/source/sourceResult?choice=Gene&option=Name&criteria=MMPL1__________-1A) | 1.60 | [2310005C01Rik](http://genome-www4.stanford.edu/cgi-bin/SMD/source/sourceResult?choice=Gene&option=Name&criteria=Cox6c__________-1A) | 1.10 | [Dct](http://genome-www4.stanford.edu/cgi-bin/SMD/source/sourceResult?choice=Gene&option=Name&criteria=Ambp___________-1A) | 1.10 | [KIAA1357](http://genome-www4.stanford.edu/cgi-bin/SMD/source/sourceResult?choice=Gene&option=Name&criteria=PRKACG_________-1A) | 1.10 |
| [1700019B01Rik](http://genome-www4.stanford.edu/cgi-bin/SMD/source/sourceResult?choice=Gene&option=Name&criteria=CPSF4__________-1A) | 1.50 | [2310035C23Rik](http://genome-www4.stanford.edu/cgi-bin/SMD/source/sourceResult?choice=Gene&option=Name&criteria=DJ971N18.2_____-1A) | 1.10 | Ddost | 1.10 | [Klf4](http://genome-www4.stanford.edu/cgi-bin/SMD/source/sourceResult?choice=Gene&option=Name&criteria=Lyst___________-1A) | 1.10 |
| [Cd68](http://genome-www4.stanford.edu/cgi-bin/SMD/source/sourceResult?choice=Gene&option=Name&criteria=Cxcl13_________-1A) | 1.50 | [2610305J24Rik](http://genome-www4.stanford.edu/cgi-bin/SMD/source/sourceResult?choice=Gene&option=Name&criteria=MOR278-1_______-1A) | 1.10 | [DHFR](http://genome-www4.stanford.edu/cgi-bin/SMD/source/sourceResult?choice=Gene&option=Name&criteria=HGAF128541_____-1A) | 1.10 | [KLF5](http://genome-www4.stanford.edu/cgi-bin/SMD/source/sourceResult?choice=Gene&option=Name&criteria=DHFR___________-1A) | 1.10 |
| [1700123D08Rik](http://genome-www4.stanford.edu/cgi-bin/SMD/source/sourceResult?choice=Gene&option=Name&criteria=Gsk3b__________-1A) | 1.40 | [3300002A11Rik](http://genome-www4.stanford.edu/cgi-bin/SMD/source/sourceResult?choice=Gene&option=Name&criteria=APOL6__________-1A) | 1.10 | [Dhx33](http://genome-www4.stanford.edu/cgi-bin/SMD/source/sourceResult?choice=Gene&option=Name&criteria=Gus____________-1A) | 1.10 | [KLRC2](http://genome-www4.stanford.edu/cgi-bin/SMD/source/sourceResult?choice=Gene&option=Name&criteria=MOR34-2________-1A) | 1.10 |
| [2410116I05Rik](http://genome-www4.stanford.edu/cgi-bin/SMD/source/sourceResult?choice=Gene&option=Name&criteria=Dhx36__________-1A) | 1.40 | [4631422O05Rik](http://genome-www4.stanford.edu/cgi-bin/SMD/source/sourceResult?choice=Gene&option=Name&criteria=RNF34__________-1A) | 1.10 | [DJ971N18.2](http://genome-www4.stanford.edu/cgi-bin/SMD/source/sourceResult?choice=Gene&option=Name&criteria=ENSG00000041951-1A) | 1.10 | [KRT13](http://genome-www4.stanford.edu/cgi-bin/SMD/source/sourceResult?choice=Gene&option=Name&criteria=Rnpc2__________-1A) | 1.10 |
| [4930428F12Rik](http://genome-www4.stanford.edu/cgi-bin/SMD/source/sourceResult?choice=Gene&option=Name&criteria=SLC38A3________-1A) | 1.30 | [4921524L21Rik](http://genome-www4.stanford.edu/cgi-bin/SMD/source/sourceResult?choice=Gene&option=Name&criteria=C:8770_________-1A) | 1.10 | DMD | 1.10 | [Krtap14](http://genome-www4.stanford.edu/cgi-bin/SMD/source/sourceResult?choice=Gene&option=Name&criteria=Heph___________-1A) | 1.10 |
| [4930519N16Rik](http://genome-www4.stanford.edu/cgi-bin/SMD/source/sourceResult?choice=Gene&option=Name&criteria=Epha2__________-1A) | 1.30 | [4930557A04Rik](http://genome-www4.stanford.edu/cgi-bin/SMD/source/sourceResult?choice=Gene&option=Name&criteria=C:3409_________-1A) | 1.10 | [DNAI1](http://genome-www4.stanford.edu/cgi-bin/SMD/source/sourceResult?choice=Gene&option=Name&criteria=Klf4___________-1A) | 1.10 | [LAIR1](http://genome-www4.stanford.edu/cgi-bin/SMD/source/sourceResult?choice=Gene&option=Name&criteria=Cacna1i________-1A) | 1.10 |
| [ADAMTS10](http://genome-www4.stanford.edu/cgi-bin/SMD/source/sourceResult?choice=Gene&option=Name&criteria=PLA2G7_________-1A) | 1.30 | [4931400O07Rik](http://genome-www4.stanford.edu/cgi-bin/SMD/source/sourceResult?choice=Gene&option=Name&criteria=LOC93064_______-1A) | 1.10 | [DNASE1L1](http://genome-www4.stanford.edu/cgi-bin/SMD/source/sourceResult?choice=Gene&option=Name&criteria=DNAI1__________-1A) | 1.10 | [LEF1](http://genome-www4.stanford.edu/cgi-bin/SMD/source/sourceResult?choice=Gene&option=Name&criteria=LRP5___________-1A) | 1.10 |
| [C:9240](http://genome-www4.stanford.edu/cgi-bin/SMD/source/sourceResult?choice=Gene&option=Name&criteria=4921524L21Rik__-1A) | 1.30 | [4931421K24Rik](http://genome-www4.stanford.edu/cgi-bin/SMD/source/sourceResult?choice=Gene&option=Name&criteria=Abca4__________-1A) | 1.10 | [DRIL1](http://genome-www4.stanford.edu/cgi-bin/SMD/source/sourceResult?choice=Gene&option=Name&criteria=Cd5____________-1A) | 1.10 | [LHFP](http://genome-www4.stanford.edu/cgi-bin/SMD/source/sourceResult?choice=Gene&option=Name&criteria=RPIP8__________-1A) | 1.10 |
| [CXCR3](http://genome-www4.stanford.edu/cgi-bin/SMD/source/sourceResult?choice=Gene&option=Name&criteria=4930519N16Rik__-1A) | 1.30 | [4932408F18Rik](http://genome-www4.stanford.edu/cgi-bin/SMD/source/sourceResult?choice=Gene&option=Name&criteria=ITGB8__________-1A) | 1.10 | [DSCR1L2](http://genome-www4.stanford.edu/cgi-bin/SMD/source/sourceResult?choice=Gene&option=Name&criteria=KIAA0748_______-1A) | 1.10 | [Limk1](http://genome-www4.stanford.edu/cgi-bin/SMD/source/sourceResult?choice=Gene&option=Name&criteria=Ahr____________-1A) | 1.10 |
| [LOC162427](http://genome-www4.stanford.edu/cgi-bin/SMD/source/sourceResult?choice=Gene&option=Name&criteria=Mrg2___________-1A) | 1.30 | [6030422M01Rik](http://genome-www4.stanford.edu/cgi-bin/SMD/source/sourceResult?choice=Gene&option=Name&criteria=2310005C01Rik__-1A) | 1.10 | [Egfl6](http://genome-www4.stanford.edu/cgi-bin/SMD/source/sourceResult?choice=Gene&option=Name&criteria=KIAA0196_______-1A) | 1.10 | [LIMS2](http://genome-www4.stanford.edu/cgi-bin/SMD/source/sourceResult?choice=Gene&option=Name&criteria=ETV7___________-1A) | 1.10 |
| [Mfn1](http://genome-www4.stanford.edu/cgi-bin/SMD/source/sourceResult?choice=Gene&option=Name&criteria=Gata1__________-1A) | 1.30 | [8430408H12Rik](http://genome-www4.stanford.edu/cgi-bin/SMD/source/sourceResult?choice=Gene&option=Name&criteria=GARP___________-1A) | 1.10 | [EMP3](http://genome-www4.stanford.edu/cgi-bin/SMD/source/sourceResult?choice=Gene&option=Name&criteria=GFI1B__________-1A) | 1.10 | LIPG | 1.10 |
| MurinemRNAforT | 1.30 | [9230102M18Rik](http://genome-www4.stanford.edu/cgi-bin/SMD/source/sourceResult?choice=Gene&option=Name&criteria=OGT____________-1A) | 1.10 | ENSG00000024600 | 1.10 | [LNK](http://genome-www4.stanford.edu/cgi-bin/SMD/source/sourceResult?choice=Gene&option=Name&criteria=C17ORF31_______-1A) | 1.10 |
| [P5cr2](http://genome-www4.stanford.edu/cgi-bin/SMD/source/sourceResult?choice=Gene&option=Name&criteria=ARPC4__________-1A) | 1.30 | [9430022F06Rik](http://genome-www4.stanford.edu/cgi-bin/SMD/source/sourceResult?choice=Gene&option=Name&criteria=MST1___________-1A) | 1.10 | ENSG00000028410 | 1.10 | [LOC151094](http://genome-www4.stanford.edu/cgi-bin/SMD/source/sourceResult?choice=Gene&option=Name&criteria=MOR155-2_______-1A) | 1.10 |
| [1500031A17Rik](http://genome-www4.stanford.edu/cgi-bin/SMD/source/sourceResult?choice=Gene&option=Name&criteria=CDT6___________-1A) | 1.20 | A:04413 | 1.10 | ENSG00000034299 | 1.10 | LOC93064 | 1.10 |
| [2700046G09Rik](http://genome-www4.stanford.edu/cgi-bin/SMD/source/sourceResult?choice=Gene&option=Name&criteria=Dap3___________-1A) | 1.20 | [Abca4](http://genome-www4.stanford.edu/cgi-bin/SMD/source/sourceResult?choice=Gene&option=Name&criteria=MMP3___________-1A) | 1.10 | ENSG00000041752 | 1.10 | [Mist1](http://genome-www4.stanford.edu/cgi-bin/SMD/source/sourceResult?choice=Gene&option=Name&criteria=FLJ10565_______-1A) | 1.10 |
| [3300001M08Rik](http://genome-www4.stanford.edu/cgi-bin/SMD/source/sourceResult?choice=Gene&option=Name&criteria=LAMC3__________-1A) | 1.20 | [ADORA2A](http://genome-www4.stanford.edu/cgi-bin/SMD/source/sourceResult?choice=Gene&option=Name&criteria=MDH2___________-1A) | 1.10 | ENSG00000041951 | 1.10 | [MMP3](http://genome-www4.stanford.edu/cgi-bin/SMD/source/sourceResult?choice=Gene&option=Name&criteria=Ddx19__________-1A) | 1.10 |
| [4930549C01Rik](http://genome-www4.stanford.edu/cgi-bin/SMD/source/sourceResult?choice=Gene&option=Name&criteria=Cyp2j6_________-1A) | 1.20 | [Ahr](http://genome-www4.stanford.edu/cgi-bin/SMD/source/sourceResult?choice=Gene&option=Name&criteria=GATM___________-1A) | 1.10 | [Epha2](http://genome-www4.stanford.edu/cgi-bin/SMD/source/sourceResult?choice=Gene&option=Name&criteria=SLC5A5_________-1A) | 1.10 | MMPL1 | 1.10 |
| [4932432N11Rik](http://genome-www4.stanford.edu/cgi-bin/SMD/source/sourceResult?choice=Gene&option=Name&criteria=D8Ertd354e_____-1A) | 1.20 | [AI159731](http://genome-www4.stanford.edu/cgi-bin/SMD/source/sourceResult?choice=Gene&option=Name&criteria=4931421K24Rik__-1A) | 1.10 | [Ethe1](http://genome-www4.stanford.edu/cgi-bin/SMD/source/sourceResult?choice=Gene&option=Name&criteria=COX5A__________-1A) | 1.10 | MOR104-4 | 1.10 |
| [Ambp](http://genome-www4.stanford.edu/cgi-bin/SMD/source/sourceResult?choice=Gene&option=Name&criteria=Gpm6a__________-1A) | 1.20 | [AI790298](http://genome-www4.stanford.edu/cgi-bin/SMD/source/sourceResult?choice=Gene&option=Name&criteria=HGS____________-1A) | 1.10 | [ETV7](http://genome-www4.stanford.edu/cgi-bin/SMD/source/sourceResult?choice=Gene&option=Name&criteria=OTOR___________-1A) | 1.10 | [MOR155-2](http://genome-www4.stanford.edu/cgi-bin/SMD/source/sourceResult?choice=Gene&option=Name&criteria=Ofd1___________-1A) | 1.10 |
| [Atp7a](http://genome-www4.stanford.edu/cgi-bin/SMD/source/sourceResult?choice=Gene&option=Name&criteria=1300017E09Rik__-1A) | 1.20 | [Akr1e1](http://genome-www4.stanford.edu/cgi-bin/SMD/source/sourceResult?choice=Gene&option=Name&criteria=Bklhd2_________-1A) | 1.10 | [F2](http://genome-www4.stanford.edu/cgi-bin/SMD/source/sourceResult?choice=Gene&option=Name&criteria=HRC____________-1A) | 1.10 | [MOR170-3](http://genome-www4.stanford.edu/cgi-bin/SMD/source/sourceResult?choice=Gene&option=Name&criteria=2610305J24Rik__-1A) | 1.10 |
| [CA3](http://genome-www4.stanford.edu/cgi-bin/SMD/source/sourceResult?choice=Gene&option=Name&criteria=ORM1___________-1A) | 1.20 | [ANK3](http://genome-www4.stanford.edu/cgi-bin/SMD/source/sourceResult?choice=Gene&option=Name&criteria=COL6A1_________-1A) | 1.10 | [FAP](http://genome-www4.stanford.edu/cgi-bin/SMD/source/sourceResult?choice=Gene&option=Name&criteria=Ldh2___________-1A) | 1.10 | [MOR278-1](http://genome-www4.stanford.edu/cgi-bin/SMD/source/sourceResult?choice=Gene&option=Name&criteria=SEC13L1________-1A) | 1.10 |
| [CCL19](http://genome-www4.stanford.edu/cgi-bin/SMD/source/sourceResult?choice=Gene&option=Name&criteria=RNF5___________-1A) | 1.20 | APBB2 | 1.10 | [FEZL](http://genome-www4.stanford.edu/cgi-bin/SMD/source/sourceResult?choice=Gene&option=Name&criteria=A:04413________-1A) | 1.10 | [MOR34-2](http://genome-www4.stanford.edu/cgi-bin/SMD/source/sourceResult?choice=Gene&option=Name&criteria=DRIL1__________-1A) | 1.10 |
| [CPSF4](http://genome-www4.stanford.edu/cgi-bin/SMD/source/sourceResult?choice=Gene&option=Name&criteria=Niban__________-1A) | 1.20 | [Apoe](http://genome-www4.stanford.edu/cgi-bin/SMD/source/sourceResult?choice=Gene&option=Name&criteria=ADORA2A________-1A) | 1.10 | [FLJ10565](http://genome-www4.stanford.edu/cgi-bin/SMD/source/sourceResult?choice=Gene&option=Name&criteria=IL23A__________-1A) | 1.10 | [Mrg2](http://genome-www4.stanford.edu/cgi-bin/SMD/source/sourceResult?choice=Gene&option=Name&criteria=LNK____________-1A) | 1.10 |
| [CR2gene](http://genome-www4.stanford.edu/cgi-bin/SMD/source/sourceResult?choice=Gene&option=Name&criteria=FRZB___________-1A) | 1.20 | [APOL6](http://genome-www4.stanford.edu/cgi-bin/SMD/source/sourceResult?choice=Gene&option=Name&criteria=4932408F18Rik__-1A) | 1.10 | [Gata1](http://genome-www4.stanford.edu/cgi-bin/SMD/source/sourceResult?choice=Gene&option=Name&criteria=COL6A1_________-1B) | 1.10 | [MST1](http://genome-www4.stanford.edu/cgi-bin/SMD/source/sourceResult?choice=Gene&option=Name&criteria=Pik3r4_________-1A) | 1.10 |
| [CTSK](http://genome-www4.stanford.edu/cgi-bin/SMD/source/sourceResult?choice=Gene&option=Name&criteria=FLJ21924_______-1A) | 1.20 | [ARPC4](http://genome-www4.stanford.edu/cgi-bin/SMD/source/sourceResult?choice=Gene&option=Name&criteria=Ggh____________-1A) | 1.10 | [GATM](http://genome-www4.stanford.edu/cgi-bin/SMD/source/sourceResult?choice=Gene&option=Name&criteria=Capn6__________-1A) | 1.10 | [MT3](http://genome-www4.stanford.edu/cgi-bin/SMD/source/sourceResult?choice=Gene&option=Name&criteria=HTR2A__________-1A) | 1.10 |
| [Cyp2j6](http://genome-www4.stanford.edu/cgi-bin/SMD/source/sourceResult?choice=Gene&option=Name&criteria=Icam4__________-1A) | 1.20 | Atm | 1.10 | [GFI1B](http://genome-www4.stanford.edu/cgi-bin/SMD/source/sourceResult?choice=Gene&option=Name&criteria=Dct____________-1A) | 1.10 | [Mtch1](http://genome-www4.stanford.edu/cgi-bin/SMD/source/sourceResult?choice=Gene&option=Name&criteria=C:2400_________-1A) | 1.10 |
| [D8Ertd354e](http://genome-www4.stanford.edu/cgi-bin/SMD/source/sourceResult?choice=Gene&option=Name&criteria=Krtap14________-1A) | 1.20 | [ATP5G3](http://genome-www4.stanford.edu/cgi-bin/SMD/source/sourceResult?choice=Gene&option=Name&criteria=Rab15__________-1A) | 1.10 | [Ggh](http://genome-www4.stanford.edu/cgi-bin/SMD/source/sourceResult?choice=Gene&option=Name&criteria=Hrg____________-1A) | 1.10 | MYO6 | 1.10 |
| [Ddx19](http://genome-www4.stanford.edu/cgi-bin/SMD/source/sourceResult?choice=Gene&option=Name&criteria=Cyp2a5_________-1A) | 1.20 | [B4galt2](http://genome-www4.stanford.edu/cgi-bin/SMD/source/sourceResult?choice=Gene&option=Name&criteria=POLD2__________-1A) | 1.10 | [GNG5](http://genome-www4.stanford.edu/cgi-bin/SMD/source/sourceResult?choice=Gene&option=Name&criteria=CGR11__________-1A) | 1.10 | [NAG18](http://genome-www4.stanford.edu/cgi-bin/SMD/source/sourceResult?choice=Gene&option=Name&criteria=4631422O05Rik__-1A) | 1.10 |
| [Dhx36](http://genome-www4.stanford.edu/cgi-bin/SMD/source/sourceResult?choice=Gene&option=Name&criteria=FLJ11827_______-1A) | 1.20 | [BC018601](http://genome-www4.stanford.edu/cgi-bin/SMD/source/sourceResult?choice=Gene&option=Name&criteria=GTPBP2_________-1A) | 1.10 | [Gpm6a](http://genome-www4.stanford.edu/cgi-bin/SMD/source/sourceResult?choice=Gene&option=Name&criteria=NSEP1__________-1A) | 1.10 | [NAP1](http://genome-www4.stanford.edu/cgi-bin/SMD/source/sourceResult?choice=Gene&option=Name&criteria=ADAMTS10_______-1A) | 1.10 |
| [FLJ12547](http://genome-www4.stanford.edu/cgi-bin/SMD/source/sourceResult?choice=Gene&option=Name&criteria=Musmacedonicusc-1A) | 1.20 | [Bklhd2](http://genome-www4.stanford.edu/cgi-bin/SMD/source/sourceResult?choice=Gene&option=Name&criteria=9430022F06Rik__-1A) | 1.10 | [Grb10](http://genome-www4.stanford.edu/cgi-bin/SMD/source/sourceResult?choice=Gene&option=Name&criteria=MOR170-3_______-1A) | 1.10 | [Niban](http://genome-www4.stanford.edu/cgi-bin/SMD/source/sourceResult?choice=Gene&option=Name&criteria=ENSG00000024600-1A) | 1.10 |
| [FLJ21924](http://genome-www4.stanford.edu/cgi-bin/SMD/source/sourceResult?choice=Gene&option=Name&criteria=4930557A04Rik__-1A) | 1.20 | [BSG](http://genome-www4.stanford.edu/cgi-bin/SMD/source/sourceResult?choice=Gene&option=Name&criteria=Irf3___________-1A) | 1.10 | [Gsto1](http://genome-www4.stanford.edu/cgi-bin/SMD/source/sourceResult?choice=Gene&option=Name&criteria=BSG____________-1A) | 1.10 | [Nolc1](http://genome-www4.stanford.edu/cgi-bin/SMD/source/sourceResult?choice=Gene&option=Name&criteria=Ndufb9_________-1A) | 1.10 |
| [FRZB](http://genome-www4.stanford.edu/cgi-bin/SMD/source/sourceResult?choice=Gene&option=Name&criteria=CLONE017D02MY02-1A) | 1.20 | [C:2400](http://genome-www4.stanford.edu/cgi-bin/SMD/source/sourceResult?choice=Gene&option=Name&criteria=Fln29__________-1A) | 1.10 | [GTPBP2](http://genome-www4.stanford.edu/cgi-bin/SMD/source/sourceResult?choice=Gene&option=Name&criteria=PMM2___________-1A) | 1.10 | [NPPB](http://genome-www4.stanford.edu/cgi-bin/SMD/source/sourceResult?choice=Gene&option=Name&criteria=HGNM_005839____-1A) | 1.10 |
| [Gsk3b](http://genome-www4.stanford.edu/cgi-bin/SMD/source/sourceResult?choice=Gene&option=Name&criteria=ENSG00000034299-1A) | 1.20 | [C:3409](http://genome-www4.stanford.edu/cgi-bin/SMD/source/sourceResult?choice=Gene&option=Name&criteria=AI790298_______-1A) | 1.10 | [Gus](http://genome-www4.stanford.edu/cgi-bin/SMD/source/sourceResult?choice=Gene&option=Name&criteria=Limk1__________-1A) | 1.10 | NSEP1 | 1.10 |
| [Icam4](http://genome-www4.stanford.edu/cgi-bin/SMD/source/sourceResult?choice=Gene&option=Name&criteria=MCP____________-1A) | 1.20 | [C:8770](http://genome-www4.stanford.edu/cgi-bin/SMD/source/sourceResult?choice=Gene&option=Name&criteria=FLJ23119_______-1A) | 1.10 | [Hand1](http://genome-www4.stanford.edu/cgi-bin/SMD/source/sourceResult?choice=Gene&option=Name&criteria=HPIP___________-1A) | 1.10 | [Ofd1](http://genome-www4.stanford.edu/cgi-bin/SMD/source/sourceResult?choice=Gene&option=Name&criteria=LHFP___________-1A) | 1.10 |
| [IL17](http://genome-www4.stanford.edu/cgi-bin/SMD/source/sourceResult?choice=Gene&option=Name&criteria=Ddost__________-1A) | 1.20 | [C11ORF17](http://genome-www4.stanford.edu/cgi-bin/SMD/source/sourceResult?choice=Gene&option=Name&criteria=Grb10__________-1A) | 1.10 | [HDLBP](http://genome-www4.stanford.edu/cgi-bin/SMD/source/sourceResult?choice=Gene&option=Name&criteria=3300002A11Rik__-1A) | 1.10 | [OGT](http://genome-www4.stanford.edu/cgi-bin/SMD/source/sourceResult?choice=Gene&option=Name&criteria=SDCCAG1________-1A) | 1.10 |
| [KIAA0196](http://genome-www4.stanford.edu/cgi-bin/SMD/source/sourceResult?choice=Gene&option=Name&criteria=POMT2__________-1A) | 1.20 | [C17ORF31](http://genome-www4.stanford.edu/cgi-bin/SMD/source/sourceResult?choice=Gene&option=Name&criteria=ATP5G3_________-1A) | 1.10 | [Heph](http://genome-www4.stanford.edu/cgi-bin/SMD/source/sourceResult?choice=Gene&option=Name&criteria=2310035C23Rik__-1A) | 1.10 | OPRK1 | 1.10 |
| [LAMC3](http://genome-www4.stanford.edu/cgi-bin/SMD/source/sourceResult?choice=Gene&option=Name&criteria=Khk____________-1A) | 1.20 | [Cd5](http://genome-www4.stanford.edu/cgi-bin/SMD/source/sourceResult?choice=Gene&option=Name&criteria=NAG18__________-1A) | 1.10 | [HGAF128541](http://genome-www4.stanford.edu/cgi-bin/SMD/source/sourceResult?choice=Gene&option=Name&criteria=COL18A1________-1A) | 1.10 | [ORM1](http://genome-www4.stanford.edu/cgi-bin/SMD/source/sourceResult?choice=Gene&option=Name&criteria=Cdc26__________-1A) | 1.10 |
| [Ldh2](http://genome-www4.stanford.edu/cgi-bin/SMD/source/sourceResult?choice=Gene&option=Name&criteria=Apoe___________-1A) | 1.20 | [Cdc26](http://genome-www4.stanford.edu/cgi-bin/SMD/source/sourceResult?choice=Gene&option=Name&criteria=Cyp2c40________-1A) | 1.10 | [HGBC004969](http://genome-www4.stanford.edu/cgi-bin/SMD/source/sourceResult?choice=Gene&option=Name&criteria=K-ALPHA-1______-1A) | 1.10 | [OTOR](http://genome-www4.stanford.edu/cgi-bin/SMD/source/sourceResult?choice=Gene&option=Name&criteria=4931400O07Rik__-1A) | 1.10 |
| [M11S1](http://genome-www4.stanford.edu/cgi-bin/SMD/source/sourceResult?choice=Gene&option=Name&criteria=CR2gene________-1A) | 1.20 | [CDT6](http://genome-www4.stanford.edu/cgi-bin/SMD/source/sourceResult?choice=Gene&option=Name&criteria=CA3____________-1A) | 1.10 | [HGNM 005839](http://genome-www4.stanford.edu/cgi-bin/SMD/source/sourceResult?choice=Gene&option=Name&criteria=ANK3___________-1A) | 1.10 | [Pabpc1](http://genome-www4.stanford.edu/cgi-bin/SMD/source/sourceResult?choice=Gene&option=Name&criteria=PDE1B__________-1A) | 1.10 |
| Muscarolicytoch | 1.20 | [CGI-69](http://genome-www4.stanford.edu/cgi-bin/SMD/source/sourceResult?choice=Gene&option=Name&criteria=ITGA9__________-1A) | 1.10 | [HGS](http://genome-www4.stanford.edu/cgi-bin/SMD/source/sourceResult?choice=Gene&option=Name&criteria=EMP3___________-1A) | 1.10 | PDE1B | 1.10 |
| Musmacedonicusc | 1.20 | [CGR11](http://genome-www4.stanford.edu/cgi-bin/SMD/source/sourceResult?choice=Gene&option=Name&criteria=ENSG00000028410-1A) | 1.10 | [HPIP](http://genome-www4.stanford.edu/cgi-bin/SMD/source/sourceResult?choice=Gene&option=Name&criteria=CLONEB320UT-CEL-1A) | 1.10 | [PHC2](http://genome-www4.stanford.edu/cgi-bin/SMD/source/sourceResult?choice=Gene&option=Name&criteria=C330018L13Rik__-1A) | 1.10 |
| [Ndufa10](http://genome-www4.stanford.edu/cgi-bin/SMD/source/sourceResult?choice=Gene&option=Name&criteria=Akr1e1_________-1A) | 1.20 | [Clca4](http://genome-www4.stanford.edu/cgi-bin/SMD/source/sourceResult?choice=Gene&option=Name&criteria=MOR104-4_______-1A) | 1.10 | [HRC](http://genome-www4.stanford.edu/cgi-bin/SMD/source/sourceResult?choice=Gene&option=Name&criteria=Cxcl15_________-1A) | 1.10 | [Pik3r4](http://genome-www4.stanford.edu/cgi-bin/SMD/source/sourceResult?choice=Gene&option=Name&criteria=Cd68___________-1A) | 1.10 |
| [Ndufb9](http://genome-www4.stanford.edu/cgi-bin/SMD/source/sourceResult?choice=Gene&option=Name&criteria=FOURANDAHALFLIM-1B) | 1.20 | CLONE017D02MY02 | 1.10 | [Hrg](http://genome-www4.stanford.edu/cgi-bin/SMD/source/sourceResult?choice=Gene&option=Name&criteria=Egfl6__________-1A) | 1.10 | PLA2G7 | 1.10 |
| [NEB](http://genome-www4.stanford.edu/cgi-bin/SMD/source/sourceResult?choice=Gene&option=Name&criteria=FAP____________-1A) | 1.20 | CLONEB320UT-CEL | 1.10 | [HRH2](http://genome-www4.stanford.edu/cgi-bin/SMD/source/sourceResult?choice=Gene&option=Name&criteria=HGBC004969_____-1A) | 1.10 | [PMM2](http://genome-www4.stanford.edu/cgi-bin/SMD/source/sourceResult?choice=Gene&option=Name&criteria=Mef2b__________-1A) | 1.10 |
| [Og9x](http://genome-www4.stanford.edu/cgi-bin/SMD/source/sourceResult?choice=Gene&option=Name&criteria=1500031A17Rik__-1A) | 1.20 | [COL18A1](http://genome-www4.stanford.edu/cgi-bin/SMD/source/sourceResult?choice=Gene&option=Name&criteria=Gsto1__________-1A) | 1.10 | [HTR2A](http://genome-www4.stanford.edu/cgi-bin/SMD/source/sourceResult?choice=Gene&option=Name&criteria=Camk2a_________-1A) | 1.10 | [POLD2](http://genome-www4.stanford.edu/cgi-bin/SMD/source/sourceResult?choice=Gene&option=Name&criteria=HDLBP__________-1A) | 1.10 |
| [ORMDL2](http://genome-www4.stanford.edu/cgi-bin/SMD/source/sourceResult?choice=Gene&option=Name&criteria=3300001M08Rik__-1A) | 1.20 | [Col4a2](http://genome-www4.stanford.edu/cgi-bin/SMD/source/sourceResult?choice=Gene&option=Name&criteria=DNASE1L1_______-1A) | 1.10 | [IL23A](http://genome-www4.stanford.edu/cgi-bin/SMD/source/sourceResult?choice=Gene&option=Name&criteria=LOC151094______-1A) | 1.10 | [POMT2](http://genome-www4.stanford.edu/cgi-bin/SMD/source/sourceResult?choice=Gene&option=Name&criteria=LEF1___________-1A) | 1.10 |
| [Ors18](http://genome-www4.stanford.edu/cgi-bin/SMD/source/sourceResult?choice=Gene&option=Name&criteria=P5cr2__________-1A) | 1.20 | [COL6A1](http://genome-www4.stanford.edu/cgi-bin/SMD/source/sourceResult?choice=Gene&option=Name&criteria=Atm____________-1A) | 1.10 | [IL24](http://genome-www4.stanford.edu/cgi-bin/SMD/source/sourceResult?choice=Gene&option=Name&criteria=PHC2___________-1A) | 1.10 | [Pou5f1](http://genome-www4.stanford.edu/cgi-bin/SMD/source/sourceResult?choice=Gene&option=Name&criteria=BC018601_______-1A) | 1.10 |
| [Ptpn12](http://genome-www4.stanford.edu/cgi-bin/SMD/source/sourceResult?choice=Gene&option=Name&criteria=LAIR1__________-1A) | 1.20 | COL6A1 | 1.10 | [Irf3](http://genome-www4.stanford.edu/cgi-bin/SMD/source/sourceResult?choice=Gene&option=Name&criteria=APBB2__________-1A) | 1.10 | [Rab15](http://genome-www4.stanford.edu/cgi-bin/SMD/source/sourceResult?choice=Gene&option=Name&criteria=Mtch1__________-1A) | 1.10 |
| [RNF5](http://genome-www4.stanford.edu/cgi-bin/SMD/source/sourceResult?choice=Gene&option=Name&criteria=1700029M23Rik__-1A) | 1.20 | [COX5A](http://genome-www4.stanford.edu/cgi-bin/SMD/source/sourceResult?choice=Gene&option=Name&criteria=ENSG00000041752-1A) | 1.10 | [ITGA9](http://genome-www4.stanford.edu/cgi-bin/SMD/source/sourceResult?choice=Gene&option=Name&criteria=NPPB___________-1A) | 1.10 | [Rab6](http://genome-www4.stanford.edu/cgi-bin/SMD/source/sourceResult?choice=Gene&option=Name&criteria=MGC14560_______-1A) | 1.10 |
| RPIP8 | 1.20 | [Cox6c](http://genome-www4.stanford.edu/cgi-bin/SMD/source/sourceResult?choice=Gene&option=Name&criteria=8430408H12Rik__-1A) | 1.10 | [ITGB8](http://genome-www4.stanford.edu/cgi-bin/SMD/source/sourceResult?choice=Gene&option=Name&criteria=LIPG___________-1A) | 1.10 | [RNF34](http://genome-www4.stanford.edu/cgi-bin/SMD/source/sourceResult?choice=Gene&option=Name&criteria=F2_____________-1A) | 1.10 |
| [SLC5A5](http://genome-www4.stanford.edu/cgi-bin/SMD/source/sourceResult?choice=Gene&option=Name&criteria=1700125F08Rik__-1A) | 1.20 | [Cxcl13](http://genome-www4.stanford.edu/cgi-bin/SMD/source/sourceResult?choice=Gene&option=Name&criteria=NAP1___________-1A) | 1.10 | [JAK1](http://genome-www4.stanford.edu/cgi-bin/SMD/source/sourceResult?choice=Gene&option=Name&criteria=Rab6___________-1A) | 1.10 | [Rnpc2](http://genome-www4.stanford.edu/cgi-bin/SMD/source/sourceResult?choice=Gene&option=Name&criteria=DSCR1L2________-1A) | 1.10 |
| [1300017E09Rik](http://genome-www4.stanford.edu/cgi-bin/SMD/source/sourceResult?choice=Gene&option=Name&criteria=DMD____________-5A) | 1.10 | Cxcl15 | 1.10 | K-ALPHA-1 | 1.10 | [SCNN1B](http://genome-www4.stanford.edu/cgi-bin/SMD/source/sourceResult?choice=Gene&option=Name&criteria=CGI-69_________-1A) | 1.10 |
| [1700029M23Rik](http://genome-www4.stanford.edu/cgi-bin/SMD/source/sourceResult?choice=Gene&option=Name&criteria=KLF5___________-1A) | 1.10 | [Cyp2a5](http://genome-www4.stanford.edu/cgi-bin/SMD/source/sourceResult?choice=Gene&option=Name&criteria=KIAA1357_______-1A) | 1.10 | [Khk](http://genome-www4.stanford.edu/cgi-bin/SMD/source/sourceResult?choice=Gene&option=Name&criteria=Atp7a__________-1A) | 1.10 | [SDCCAG1](http://genome-www4.stanford.edu/cgi-bin/SMD/source/sourceResult?choice=Gene&option=Name&criteria=IL17___________-1A) | 1.10 |
| [1700125F08Rik](http://genome-www4.stanford.edu/cgi-bin/SMD/source/sourceResult?choice=Gene&option=Name&criteria=MGC2594________-1A) | 1.10 | [Cyp2c40](http://genome-www4.stanford.edu/cgi-bin/SMD/source/sourceResult?choice=Gene&option=Name&criteria=Ethe1__________-1A) | 1.10 | [KIAA0748](http://genome-www4.stanford.edu/cgi-bin/SMD/source/sourceResult?choice=Gene&option=Name&criteria=Clca4__________-1A) | 1.10 | SEC13L1 | 1.10 |
| [1810011E08Rik](http://genome-www4.stanford.edu/cgi-bin/SMD/source/sourceResult?choice=Gene&option=Name&criteria=JAK1___________-1A) | 1.10 | [Dap3](http://genome-www4.stanford.edu/cgi-bin/SMD/source/sourceResult?choice=Gene&option=Name&criteria=B4galt2________-1A) | 1.10 | [KIAA1324](http://genome-www4.stanford.edu/cgi-bin/SMD/source/sourceResult?choice=Gene&option=Name&criteria=HRH2___________-1A) | 1.10 | [SLC38A3](http://genome-www4.stanford.edu/cgi-bin/SMD/source/sourceResult?choice=Gene&option=Name&criteria=KLRC2__________-1A) | 1.10 |
